# Constructing a novel mitochondrial metabolism-related genes signature to evaluate tumor immune microenvironment and predict survival of colorectal cancer

**DOI:** 10.3389/fmed.2025.1618471

**Published:** 2025-07-08

**Authors:** Hou Wang, Kai Zhang, Guang Ning

**Affiliations:** ^1^Department of Endocrine and Metabolic Diseases, Shanghai Institute of Endocrine and Metabolic Diseases, Ruijin Hospital, Shanghai Jiao Tong University School of Medicine, Shanghai, China; ^2^Key Laboratory for Endocrine and Metabolic Diseases of the National Health Commission of the PR China, Shanghai National Clinical Research Center for Metabolic Diseases, Shanghai National Center for Translational Medicine, Ruijin Hospital, Shanghai Jiao Tong University School of Medicine, Shanghai, China; ^3^Department of Surgery, Shanghai Key Laboratory of Gastric Neoplasms, Shanghai Institute of Digestive Surgery, Ruijin Hospital, Shanghai Jiao Tong University School of Medicine, Shanghai, China

**Keywords:** colorectal cancer, prognostic biomarker, mitochondrial metabolism, tumor microenvironment, immunotherapy, drug susceptibility

## Abstract

**Background:**

Colorectal cancer (CRC) is a highly lethal gastrointestinal malignancy with substantial global health implications. Although mitochondrial metabolism genes play a crucial role in CRC development, their prognostic significance remains unclear.

**Methods:**

This study systematically analyzed the expression and prognostic value of mitochondrial metabolism-related genes in CRC patients, establishing a risk model using data from TCGA and GEO databases. We also investigated the tumor microenvironment (TME), immune cell infiltration, tumor mutation burden, microsatellite instability (MSI), and drug sensitivity. Core mitochondrial metabolism-related gene, TMEM86B was identified and its functions validated through cell-based assays and *in vivo* mouse models.

**Results:**

Fifteen mitochondrial metabolism-related genes were identified, including HSD3B7, ORC1, GPSM2, NDUFA4L2, CHDH, LARS2, TMEM86B, FABP4, TNFAIP8L3, HMGCL, GDE1, ACOX1, ARV1, HDC, and GSR. The nomogram, which incorporates independent prognostic genes TMEM86B, TNFAIP8L3, HDC, and key clinical features pTNM stage (pathological Tumor-Node-Metastasis), age, was created to predict patient outcomes. Notable differences in immune cell infiltration were observed between risk groups. The risk score was associated with TME genes and immune checkpoints, indicating an immunosuppressive environment in high-risk groups. Furthermore, TIDE analysis revealed that integrating the risk score with immune score, stromal score, or microsatellite status improved the prediction of immunotherapy response across different CRC patient subgroups. Core mitochondrial metabolism-related gene, TMEM86B promotes colorectal cancer progression by enhancing cell proliferation, migration, and invasion, and its downregulation significantly inhibits tumor growth both *in vitro* and *in vivo*.

**Conclusion:**

Our findings indicate that the risk model associated with mitochondrial metabolism may serve as a dependable prognostic indicator, facilitating tailored therapeutic strategies for CRC patients. TMEM86B promotes colorectal cancer progression, and its downregulation inhibits tumor growth *in vitro* and *in vivo*.

## Graphical abstract

Integrated workflow and key findings. 
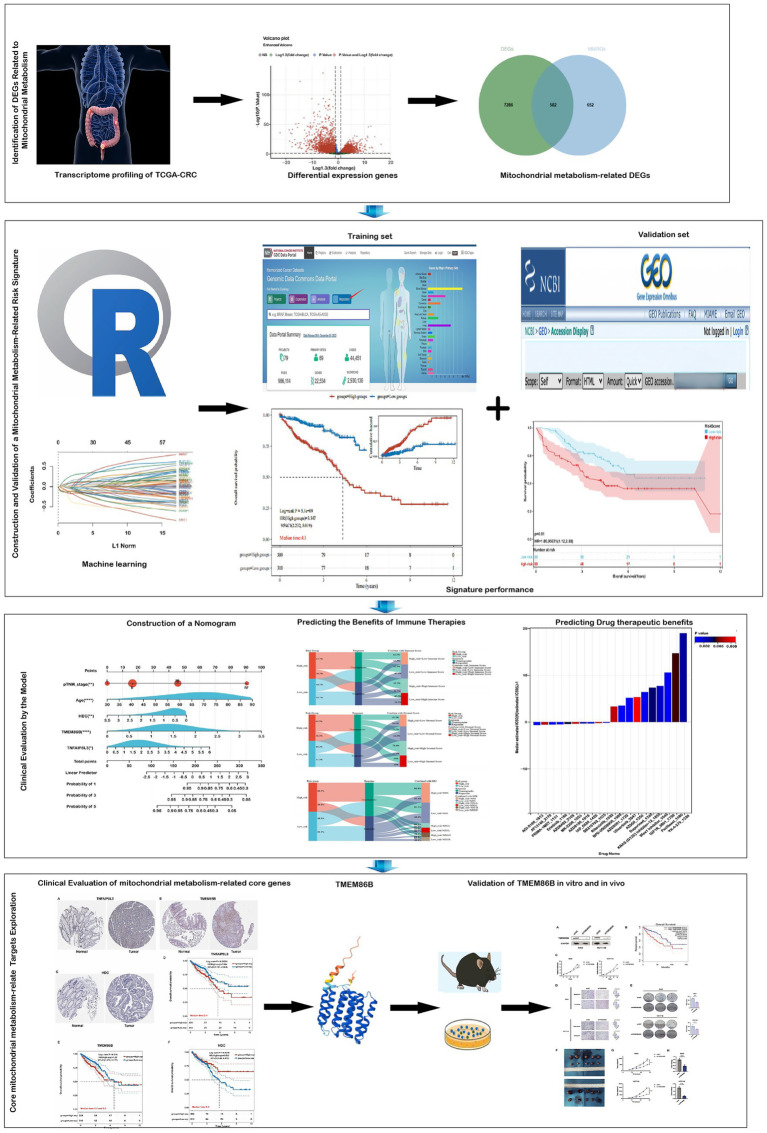

## Introduction

Colorectal cancer (CRC) was the third most commonly diagnosed cancer, with around 1.9 million new cases globally, representing 9.6% of all cancer diagnoses. Additionally, CRC was the second leading cause of cancer-related deaths, responsible for approximately 0.9 million fatalities, or 9.3% of all cancer deaths worldwide (1). Geographically, CRC incidence and mortality rates are rising swiftly in many low- and middle-income nations, while highly developed countries tend to exhibit stabilizing or declining trends, although their rates remain among the highest worldwide (2). Patients with metastatic CRC face a much poorer prognosis, with a 5-year survival rate below 20% (3). Therefore, it is crucial to identify more reliable biomarkers for prognosis prediction, and to explore potential therapeutic targets in colorectal carcinogenesis.

Mitochondrial metabolism has emerged as a promising approach for developing new anticancer therapies, with various strategies currently under investigation (4, 5). Otto Warburg observed that cancer cells favor glycolysis over oxidative phosphorylation, even in the presence of oxygen, resulting in excess lactate production, a phenomenon termed “aerobic glycolysis” or the “Warburg effect” (6, 7). Subsequent studies have demonstrated that mitochondrial function alterations, such as changes in mitochondrial biogenesis, dynamics, and metabolism, play a crucial role in tumorigenesis, progression, and resistance to therapy (8). Mitochondrial metabolism crucial role in every stage of cancer development, which ranging from malignant transformation to tumor progression and therapy response has now been acknowledged (9, 10). In fact, research has shown that increased mitochondrial metabolism can compensate for the absence of the Warburg effect in promoting the growth of B16 melanoma tumors (11). Moreover, mitochondrial metabolism is essential for tumorigenesis in Kras-driven mouse models of lung adenocarcinoma (12).

Mitochondrial metabolism plays a critical role in shaping the tumor microenvironment (TME) and influencing colorectal cancer progression. Altered mitochondrial function supports tumor growth and metastasis by adapting metabolic and genetic responses to TME changes (13). Damaged mitochondria release ROS, mtDNA, and mtDAMPs under stress, activating immune pathways and triggering T-cell responses (14–16). However, increased mitochondrial oxidative phosphorylation (OXPHOS) can worsen tumor hypoxia, promoting immunosuppression and reducing the effectiveness of therapies like anti-PD-1 (17, 18). This immunosuppressive TME favors immune evasion by promoting M2 macrophages and regulatory T cells (Tregs) (19), while mitochondrial alterations can also upregulate PD-1/PD-L1 pathways, further diminishing immunotherapy response (20). These findings highlight the close link between mitochondrial metabolism and immune suppression in the CRC microenvironment.

Recognizing the importance of mitochondrial metabolism in tumor development, the identification of biomarkers related to mitochondrial metabolism for CRC prognosis represents a promising area of research. Although multiple studies have formulated models to predict patient survival in CRC (21, 22), few of them focused on establishing prognostic models for CRC linked to mitochondrial metabolism, which effectively predicts prognosis and immunotherapy responsiveness in patients with colorectal adenocarcinoma and rectal adenocarcinoma. We further analyzed the relationship between risk scores and TME characteristics, including immune cell infiltration, immune checkpoint expression, and responses to immunotherapy. Additionally, we assessed the drug sensitivity of patients to 198 drugs. In summary, our mitochondrial metabolism-related risk model serves as a reliable prognostic biomarker for CRC, offering potential guidance for personalized treatment strategies. By linking mitochondrial metabolism to the immunosuppressive TME, this model enhances our understanding of CRC pathogenesis and paves the way for improved therapeutic interventions.

## Materials and methods

### Data collection

RNA-seq data and microsatellite status for 620 COADREAD samples were retrieved from the TCGA database.^1^ Clinical data were obtained from UCSC Xena.^2^ In total, 671 samples were analyzed, including 620 tumor samples and 51 healthy samples. Validation cohorts included GSE17536 which comprised 177 samples. Levels of the 15 Prognosis-Related genes were validated in the GSE39582 Cohort, which comprised 585 samples. To eliminate batch effects, we used the removeBatchEffect function from the limma package in R. Mitochondrial metabolism-related genes were sourced from the MSigDB database.^3^ This gene set was curated through a combination of literature review and experimental validation, and it encompasses genes involved in mitochondrial metabolic pathways (23, 24).

### Construction and validation of prognostic mitochondrial metabolism-related risk score signature

Differentially expressed genes (DEGs) between normal and tumor samples, as well as between high- and low-risk groups in the training set, were identified using the “limma” R package, with criteria of |log2 fold change| > 1.3 and adjusted *p*-value < 0.05. Volcano plots and Venn diagrams visualized these DEGs and their overlap with mitochondrial metabolism-related genes, resulting in 582 differentially expressed mitochondrial metabolism-related genes.

Univariate Cox regression analysis identified 65 of these genes as significantly associated with colorectal cancer prognosis. To construct a robust prognostic model based on mitochondrial metabolism-related genes, we applied the Least Absolute Shrinkage and Selection Operator (LASSO) regression for feature selection. A 10-fold cross-validation approach was employed to identify the optimal penalty parameter (*λ*) by minimizing the partial likelihood deviance, thereby reducing the risk of overfitting. The selected λ corresponded to the model with the lowest cross-validation error, ensuring optimal predictive performance. By shrinking regression coefficients toward zero with increasing λ, the LASSO algorithm retains only the most informative features, thus simplifying the model and enhancing its generalizability.

The risk score for each sample was calculated as:


$$\text{Risk score}=\sum {\text{expgenei}}^{*}\mathrm{\beta i}$$

where expgene, i, and βi represent the expression level of gene, the number of signature genes, and the coefficient index, respectively. Samples were then classified into high- and low-risk groups based on the median risk score. Clinical data, including gender, age, and TNM stage, were obtained from TCGA. Both univariate and multivariate Cox regression analyses confirmed that the risk score independently predicted prognosis (*p* < 0.05). To evaluate the reliability and applicability of the constructed risk model, the signature’s performance was validated in the external cohort GSE17536 and assessed using ROC curves, risk plots, and the concordance index (C-index). Gene details were sourced from the National Center for Biotechnology Information (NCBI).

### Construction and validation of nomogram

Risk scores and clinical factors (age, gender, pTNM stage) were analyzed using univariate Cox regression to identify survival-related factors (*p* < 0.05). Multivariate Cox regression identified significant survival predictors (*p* < 0.05). Based on these predictors, nomograms were constructed, assigning scores to each variable. The total score for each patient was calculated by summing the scores of the predictors in the nomogram. Patient survival outcomes at 1, 3, and 5 years were estimated using the total score and corresponding probability of survival. The nomogram model’s discrimination and accuracy were evaluated using ROC curves, calibration curves.

### Gene ontology (GO) and Kyoto encyclopedia of genes and genomes (KEGG) analyses

This study used the R packages “clusterProfiler,” “org.Hs.eg.db,” “enrichplot,” and “ggplot2” (R version 4.3.3) to analyze the functions of mitochondrial metabolism-related DEGs and DEGs between high- and low-risk groups, conduct enrichment analysis, and visually represent GO and KEGG data. An adjusted *p*-value < 0.05 was used to filter significant functional candidates.

### Gene set enrichment analyses (GSEA)

Curated sets v7.4 collections from the MSigDB were used for GSEA, performed with GSEA 4.2.1 software. The total transcriptome of tumor samples was analyzed.

### Tumor microenvironment

Stromal scores and immune scores were calculated using the ESTIMATE algorithm in R (version 4.3.3) “estimate” package. The TME-related biomarker list was extracted from GSEA.^4^ RNA-sequencing expression (level 3) profiles and clinical information for COADREAD were downloaded from the TCGA dataset.^5^ To ensure reliable immune score evaluation results, the immuneeconv R package was used, integrating EPIC and quanTIseq algorithms, each with unique advantages.

### Prediction of therapeutic sensitivity in patients with different risk scores

This study assessed the risk score’s ability to predict responses to immunotherapy and 198 chemotherapeutic/targeted drugs. The IC50 values for these drugs were calculated using the “oncopredict” package in R (version 4.3.3) and normalized. Predicted chemotherapeutic responses were based on the GDSC database.^6^ Potential immunotherapy response was inferred using the TIDE score.^7^

### Mutation analysis

Somatic mutation data were downloaded from the cBioPortal database.^8^ The “maftools” R package (version 3.5.1) was used to create a waterfall plot illustrating the mutation landscape in high- and low-risk COADREAD patients and to calculate the TMB score for each sample. The microsatellite instability (MSI) data for COADREAD in the TCGA (The Cancer Genome Atlas) project can be downloaded and accessed from the TCGA Data Portal. These data are typically stored in the Genomic Data Commons (GDC) data portal of TCGA.

### Ethics approval statement

Our study was approved by an institutional review board from the Human Research Ethics Committee of Ruijin Hospital (approval no. 2020-115) and conducted in accordance with ethical guidelines (Declaration of Helsinki). Animal experiments were approved by the local Laboratory Animal Ethics Committee of Ruijin Hospital and conducted in accordance with animal use guidelines.

### Cell lines and culture

Human colorectal cancer (CRC) cell lines RKO and HCT116 were were purchased from the American Type Culture Collection (ATCC, United States), cells were authenticated by STR profiling and free of mycoplasma contamination. Cells were cultured in DMEM medium (Meilunbio, Dalian, China) containing 10% fetal bovine serum (Gibco, Grand Island, NY, United States), 100 U mL − 1 of penicillin, and 100 μg mL − 1 of streptomycin, in a humidity culture incubator at 37°C with 5% CO2.

### Lentiviral-mediated knockdown of TMEM86B

For the knockdown of TMEM86B, target shRNA sequences (5’-3’GAAGACGTTTGAGGACGATTT) were subcloned into pGreenPuro (CMV) vector. For shRNA lentivirus infection, target cells were seeded in a 6-well plate 24 h before infection and were grown to 60–80% confluency upon transduction. Culture medium was removed, and cells were incubated with virus supernatant along with 10 μg/mL polybrene (Sigma) overnight. Virus-containing medium was replaced with fresh medium. Puromycin (Sigma) (10 μg/mL) was applied to kill non-infected cells 48 h after infection to produce stably transfected cells (RKO/shTMEM86B, HCT116/shTMEM86B).

### Western blotting

Cell sample were washed 3 times with 1 × PBS and protein extracts were prepared in RIPA cell lysis buffer (Kangwei, Beijing, China) supplemented with phosphatase inhibitor Cocktail III (Roche). The concentration of protein sample was quantified by using bicinchoninic acid protein assay kit (Pierce, Rockford, IL, United States) against a bovine serum albumin standard curve. A total of 20 μg of protein was loaded onto a 10% sodium dodecyl sulfate polyacrylamide gel and transferred onto 0.22 μm PVDF membranes (Millipore, MA, United States). The membranes were then blocked with 1 × TBST buffer containing 5% nonfat milk and incubated with corresponding antibodies at 4°C overnight. Anti-GAPDH (CAT# HRP-60,004, use a concentration of 0.02 μg/mL) was purchased from Proteintech (Rosemont, IL, United States). Anti-TMEM86B (H00255043-D01P) was purchased from Thermo Fisher Scientific. Membranes were then exposed to HRP-conjugated secondary antibody (32,460, Thermo Fisher Scientific) and developed with Thermo Pierce chemiluminescent (ECL) Western Blotting Substrate (Thermo, Waltham, MA, United States). Membranes were imaged with Tanon 5,200 system (Tanon, Shanghai, China).

### Proliferation and clone formation assays

CRC cells were seeded in 96-well plates at a density of 1,000/well (200 μL/well). A cell proliferation assay was conducted utilizing the cell counting kit8 (Dojindo, Kumamoto, Japan) according to the manufacture’s protocol. After being incubated with 20 μL of CCK-8 reagent for 2 h, OD450 was then measured by spectrophotometry (BioTek, Vermont, United States). For colony formation assay, cells were seeded at a density of 1,000 cells/well into 6-well plates and incubated at 37°C for 10 days. Cells were then washed twice with PBS and fixed in 100% methanol for 15 min, prior to staining with Giemsa solution for 20 min. The number of colonies containing ≥50 cells were counted under a microscope (IX71). All experiments were performed in triplicate.

### Migration and invasion assays

For the migration assay, cells were suspended in serum-free medium (1 × 105 cells/insert) and added to the upper chamber of the 24-well insert (membrane pore size, 8 μm; Corning Life Sciences, MA, United States). Medium containing 10% serum was added to the lower chamber. After incubation for 12 h, the cells that migrated to the bottom of the membranes were fixed and stained with 0.1% crystal violet for 30 min. For the invasion assay, chamber membranes were coated with diluted Matrigel (BD Bioscience, San Jose, CA, United States). After incubation for 24 h, the cells that invaded to the bottom of the membrane were fixed and stained with 0.1% crystal violet for 30 min. The stained cells were counted using a microscope and photographed. Ten fields were randomly selected to count, and the average number was presented.

### *In vivo* tumorigenicity assay

Male BALB/c nude mice (4–6 weeks old, purchased from SPF (Beijing) Biotechnology Co., Ltd., Beijing, China) were housed in a specific pathogen-free room in the Animal Experimental Center, Ruijin Hospital, Shanghai Jiao Tong University School of Medicine, China. Animal experiments were performed in accordance with the animal research principles and the Institution’s guidelines. Five mice were used per group (*n* = 5), based on preliminary data showing >80% statistical power (*α* = 0.05) to detect differences in tumor growth with acceptable variability (CV < 30%). This group size balances statistical reliability with ethical considerations under the 3Rs principle and is consistent with prior studies using similar xenograft models. A single-blind design was applied, in which the personnel measuring tumor size and weight were blinded to group assignments to minimize measurement bias.

Mice were subcutaneously injected with 2 × 10^6^ tumor cells (RKO/shTMEM86B and HCT116/shTMEM86B) suspended in 150 μL PBS (five mice per group). Tumor length (L) and width (W) were measured every 4 days using digital Vernier caliper. Tumor volume was determined using the following formula: volume = Length × Width2/2. All mice were sacrificed under general anesthesia 4 weeks after injection. Tumor grafts were weighed and observed systematically.

### RNA extraction and qRT-PCR assays

Tumor tissues and matched adjacent normal tissues from patients with colorectal cancer (CRC) were obtained from Ruijin Hospital (Shanghai, China). Total RNA was isolated using TRIzol reagent (Invitrogen, United States) according to the manufacturer’s protocol. Complementary DNA (cDNA) was synthesized from 1 μg of total RNA using the HiScript III RT SuperMix for qPCR (+gDNA wiper) (Vazyme, #R323). Quantitative real-time PCR (qRT-PCR) was conducted using ChamQ SYBR Color qPCR Master Mix (Vazyme, China) on a standard real-time PCR system. Relative gene expression levels were calculated using the 2^−ΔΔCt method, with 18S rRNA used as an internal reference. The primer sequences used for qRT-PCR are provided in Supplementary Table 4.

### Statistical analysis

Statistical analyses were performed using R (version 4.3.3) and GraphPad Prism 10 software. A Student’s t-test analyzed the expression and distribution of risk scores, stromal scores, immune scores, tumor purity, and TMB in different groups. The Chi-square test evaluated differences in immunotherapy response and clinical factors across groups. Correlation analyses were conducted using the Spearman method. The concordance index (C-index) estimated the predictive power of age and risk score for overall survival (OS). Univariate and multivariate Cox regression analyses estimated the predictive power of mitochondrial metabolism-related genes and clinical characteristics. For GO and KEGG enrichment analyses, *p*-values were adjusted using the Benjamini-Hochberg method, and the results were reported as false discovery rate (FDR)-adjusted *p*-values. For GSEA, normalized enrichment scores (NES) and FDR q-values were employed to assess significance, with an FDR < 0.25 considered statistically significant in accordance with standard GSEA criteria. For immune cell infiltration comparisons, FDR correction was applied to the *p*-values when comparing immune cell populations between the high- and low-risk groups. For genome-wide survival screening, *p*-values from univariate Cox regression analyses were adjusted using the Benjamini-Hochberg method to control the false discovery rate (FDR). For survival analysis of selected candidate genes or model components, raw *p*-values were reported without multiple testing correction. A *p*-value < 0.05 was considered statistically significant.

## Results

### Identification of DEGs related to mitochondria and functional enrichment analysis in COADREAD

**Figure 1:**
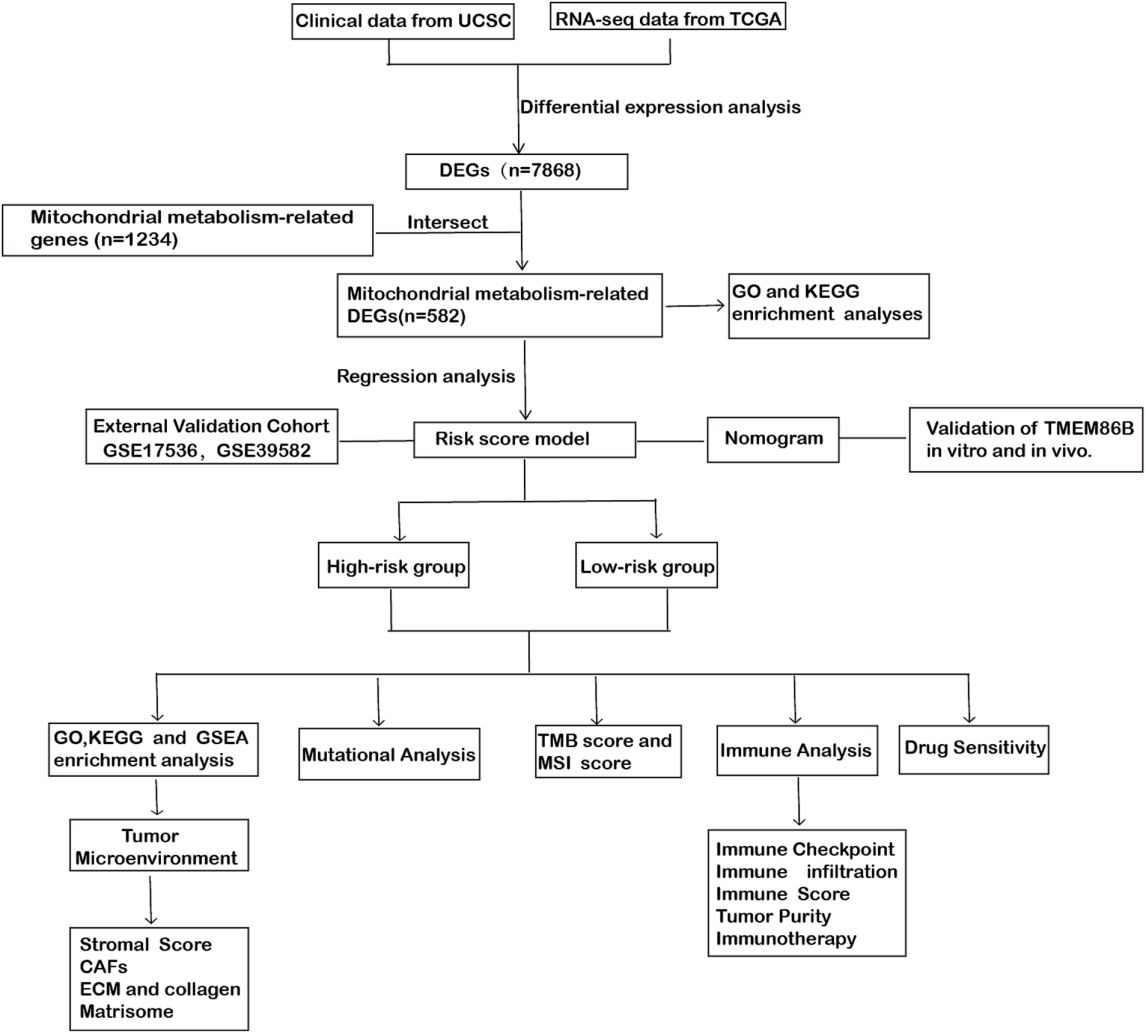
Workflow diagram: the flowchart of this study.

**Figure 2:**
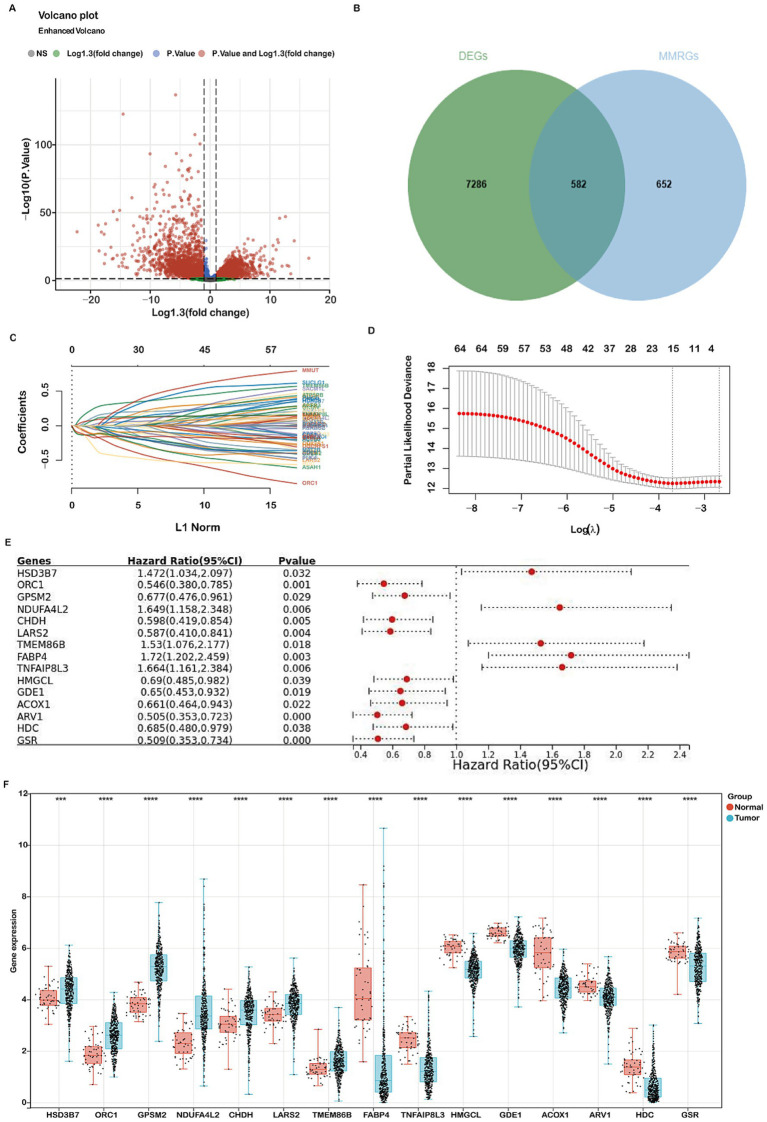
Identification of differentially expressed genes (DEGs) related to mitochondria metabolism and construction of a prognostic risk model using the TCGA-COADREAD cohort. **(A)** Volcano plot displaying 7,868 DEGs between COADREAD tumor and normal groups. **(B)** Venn diagram illustrating the overlap of 7,868 DEGs and 1,234 mitochondrial genes, resulting in the identification of 582 hub genes. **(C,D)** LASSO regression of the 65 overall survival (OS)-related genes, with cross-validation in the LASSO regression model to select the tuning parameter. The x-axis represents the log (*λ*) value, and the y-axis represents partial likelihood deviance. The red dots indicate partial likelihood deviations ± standard error for various tuning parameters. **(E)** Forest plot assessing 15 prognosis-related genes in predicting the prognosis of COADREAD, revealing their association with patient prognosis. **(F)** Gene expression levels of the 15 prognosis-related genes in the TCGA-COADREAD cohort (tumor samples: *n* = 620; normal samples: *n* = 51). *p*-values are indicated as: ***p < 0.001, **p < 0.01, *p < 0.05.

In this study, we undertook a comprehensive analysis to identify differentially expressed genes (DEGs) related to mitochondrial metabolism in COADREAD. The overall workflow is illustrated in Figure 1. We identified a total of 7,868 DEGs, using volcano plots for visualization to compare normal and tumor samples (Figure 2A). The mitochondrial metabolism gene set was derived from the MSigDB database and curated through literature review and experimental validation, encompassing genes involved in mitochondrial metabolic pathways (23, 24). From these DEGs, we further narrowed down to 582 genes related to mitochondrial metabolism by integrating genes selected from the Gene Set Enrichment Analysis (GSEA) with our identified DEGs (Figure 2B).

Gene Ontology (GO) enrichment analysis revealed significant involvement of these DEGs in various biological processes, cellular components, and molecular functions. The analysis highlighted their roles in small molecule catabolic processes and the regulation of mitochondrial components. In terms of biological processes, these DEGs were predominantly linked to small molecule metabolic processes and lipid metabolic processes (Supplementary Figure S1A). For cellular components, they were mainly associated with the mitochondrial matrix and inner membrane (Supplementary Figure S1B). Regarding molecular functions, the DEGs were significantly involved in catalytic binding and anion binding (Supplementary Figure S1C).

Additionally, KEGG pathway analysis identified several critical pathways in which these DEGs play a significant role, such as metabolic pathways, glycerophospholipid metabolism, purine metabolism, and carbon metabolism (Supplementary Figure S1D). These findings provide a comprehensive understanding of the molecular mechanisms and pathways through which mitochondrial metabolism-related DEGs contribute to the development and progression of COADREAD.

### Construction and validation of a mitochondrial metabolism-related risk signature

To develop a robust mitochondrial metabolism-related prognostic signature for colorectal cancer (COADREAD), we first performed univariate Cox regression analysis to identify 65 differentially expressed genes (DEGs) significantly associated with overall survival (*p* < 0.05; Supplementary Figure S2). These candidate prognostic genes were subsequently subjected to feature selection using the Least Absolute Shrinkage and Selection Operator (LASSO) regression algorithm (Figures 2C,D). A 10-fold cross-validation procedure was employed to avoid overfitting and determine the optimal regularization parameter. The value of *λ* corresponding to the minimum partial likelihood deviance (lambda.min=0.0246) was selected as the optimal penalty parameter for model construction. Each patient’s risk score was calculated using the following formula:


$$\begin{array}{c} \text{Risk score}=(0.1147)\times \mathrm{HSD}3\mathrm{B7}+(-0.13)\times \mathrm{ORC1}+(-0.0259)\\ \times \mathrm{GPSM2}+(0.012)\times \text{NDUFA}4\mathrm{L2}+(-0.0317)\times \text{CHDH}\\ +(-0.1246)\times \mathrm{LARS2}+(0.2367)\times \text{TMEM}8\mathrm{6B}+(0.0779)\\ \times \mathrm{FABP4}+(0.0759)\times \text{TNFAIP}8\mathrm{L3}+(-0.0059)\times \text{HMGCL}\\ +(-0.1341)\times \mathrm{GDE1}+(-0.1125)\times \mathrm{ACOX1}+(-0.0459)\\ \times \mathrm{ARV1}+(-0.2789)\times \mathrm{HDC}+(-0.1676)\times \mathrm{GSR} \end{array}$$

The prognostic value of the 15 mitochondrial metabolism-related genes in CRC was evaluated using a forest plot (Figure 2E). These genes constitute the foundation of our prognostic model, with detailed information provided in Supplementary Tables 1, 3. Analysis of the TCGA-COADREAD dataset revealed significant upregulation of GPSM2, HSD3B7, ORC1, NDUFA4L2, CHDH, LARS2, and TMEM86B, while ACOX1, ARV1, GSR, GDE1, FABP4, HDC, HMGCL, and TNFAIP8L3 were downregulated (Figure 2F).

**Figure 3:**
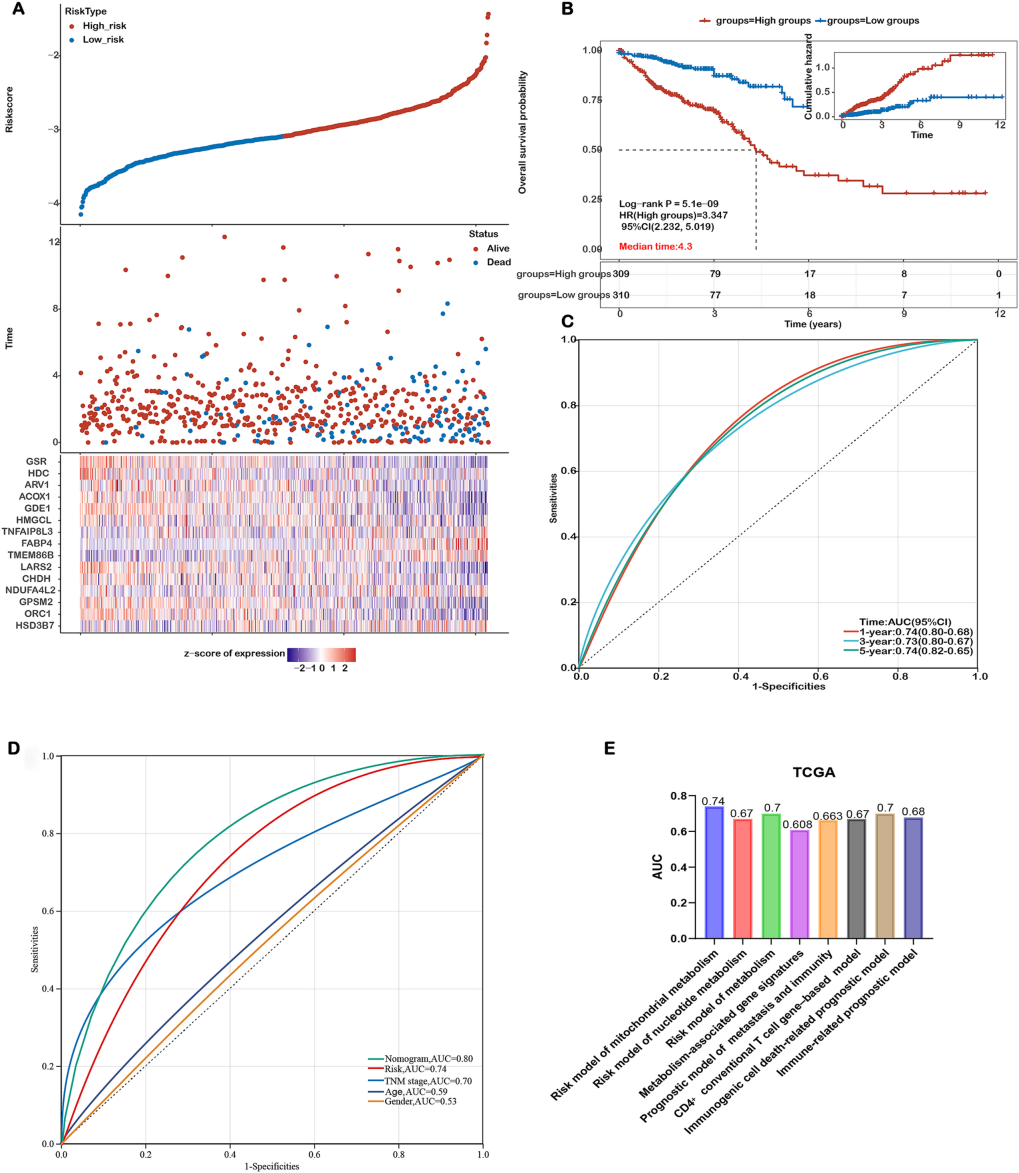
Assessing the Performance of the Prognostic Risk Model in the Training Cohort. **(A)** Distribution of risk scores, survival status (blue dots indicate deceased, red dots indicate alive), and gene expression of the 15 model genes in the TCGA-COADREAD training cohort. **(B)** Kaplan–Meier curves of overall survival (OS) for patients in the high- and low-risk groups in the TCGA-COADREAD training cohort. **(C)** ROC curves for predicting 1-, 3-, and 5-year OS in the TCGA-COADREAD training cohort. **(D)** Comparison of the risk score model, nomogram and clinicopathological characteristics in predicting the 5-year OS. **(E)** Comparison of gene expression-based prognostic signatures in CRC. Time-dependent ROC analysis for predicting overall survival outcomes at 5 years.

The relationship between risk score and survival time, survival status, risk ranking, and a heatmap of the 15 gene expression levels are depicted in Figure 3A. Patients were categorized into high-risk and low-risk subgroups based on the median risk score. Kaplan–Meier curves demonstrated significantly poorer overall survival (OS) for patients in the high-risk group (*p* = 5.1e-09, Figure 3B). The prognostic model’s predictive accuracy for 1-, 3-, and 5-year OS was evaluated using ROC curves, yielding AUC values of 0.74, 0.73, and 0.74, respectively (Figure 3C). Comparative evaluations were conducted to assess the predictive performance of our model relative to established pathological prognostic factors such as TNM staging, age, and gender (Figure 3D). The mitochondrial metabolism-related gene signature significantly outperformed traditional prognostic indicators (TNM staging, age, and gender) in terms of predictive accuracy (AUC: 0.74 vs. 0.70/0.59/0.53, respectively) and showed better stratification for 5-year overall survival (Figure 3D). These findings confirm the robustness of our risk model in predicting the prognosis of COADREAD patients.

To evaluate the prognostic performance of our 15-gene mitochondrial metabolism–related risk model, we conducted a comparative analysis with several existing CRC prognostic models based on different biological features. As shown in Figure 3E, our model achieved the highest AUC value of 0.74, surpassing the models based on nucleotide metabolism (AUC=0.67), general metabolism-related genes (AUC=0.70), immunogenic cell death–associated signatures (AUC=0.70), immune-related markers (AUC=0.678), CD4⁺ conventional T cell genes (AUC=0.67), and combined metastasis and immune gene signatures (AUC=0.663). These results indicate that our signature offers improved prognostic discrimination compared with other published models.

**Figure 4:**
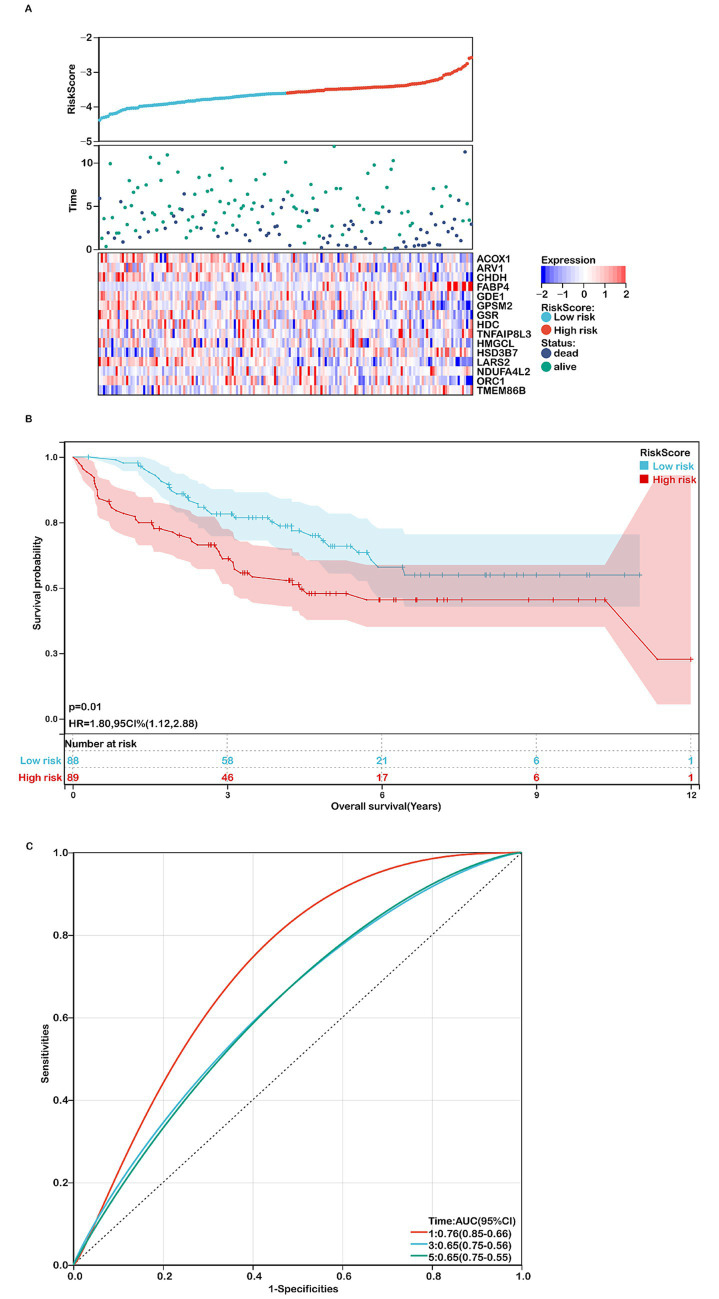
Assessing the performance of the prognostic risk model in the validation cohort. **(A)** Distribution of risk scores, survival status (red dots indicate deceased, blue dots indicate alive), and gene expression of the 15 model genes in the GSE17536 validation cohort. **(B)** Kaplan–Meier curves of overall survival (OS) for high- and low-risk groups in the GSE17536 validation cohort (*n*=177). **(C)** ROC curves for predicting 1-, 3-, and 5-year OS in the GSE17536 validation cohort.

The prognostic model was further validated using the GSE17536 dataset. Consistent with the TCGA-COADREAD cohort, higher risk scores were associated with poorer survival (Figures 4A,B). Heatmaps of the 15 gene expression levels are shown in Figure 4A. The ROC curves yielded AUCs of 0.76, 0.65, and 0.65 for 1-, 3-, and 5-year survival, respectively (Figure 4C). Similar expression patterns were observed in the GSE39582 dataset, where GPSM2, HSD3B7, ORC1, NDUFA4L2, CHDH, and LARS2 were upregulated, while ACOX1, ARV1, GSR, GDE1, FABP4, and TNFAIP8L3 were downregulated in COADREAD samples (Supplementary Figure S3). Additionally, higher risk scores correlated with more advanced T, N, M, and TNM stages (Supplementary Figure S4). However, no significant differences in other clinical characteristics were observed between high- and low-risk groups (Supplementary Table 2).

### Construction of a nomogram

A nomogram integrating independent prognostic genes (TMEM86B, TNFAIP8L3, HDC) and clinical factors (pTNM stage, age) was constructed to quantitatively predict patient outcomes and support clinical decision-making (Supplementary Figure S5C). Univariate and multivariate analyses confirmed these variables as independent prognostic factors (Supplementary Figures S5A,B). The nomogram achieved AUCs of 0.78, 0.79, and 0.80 for 1-, 3-, and 5-year overall survival, respectively (Supplementary Figure S5D), with calibration curves showing strong agreement between predicted and observed survival (Supplementary Figure S5E). The C-index was 0.757 (95% CI: 0.708–0.805, *p* = 6.272e-25). Compared to traditional prognostic indicators such as TNM stage (AUC = 0.70), age (0.59), and gender (0.53), the nomogram demonstrated superior predictive performance (AUC = 0.80; Figure 3D).

Tumor samples exhibited higher expressions of TMEM86B, lower expressions of HDC and TNFAIP8L3 compared to normal tissues (Figure 2F). Immunohistochemistry data from the HPA database corroborated these findings, showing upregulation of TMEM86B and downregulation of HDC and TNFAIP8L3 in CRC tissues compared to non-cancerous tissues (Supplementary Figures S6A–C). Kaplan–Meier analysis revealed that patients with higher expression levels of TMEM86B (*p* = 0.00551) and TNFAIP8L3 (*p* = 0.018) had shorter OS, while higher levels of HDC were associated with longer OS (*p* = 0.037, Supplementary Figures S6D–F).

### Functional enrichment analysis of DEGs in high-risk and low-risk groups

**Figure 5:**
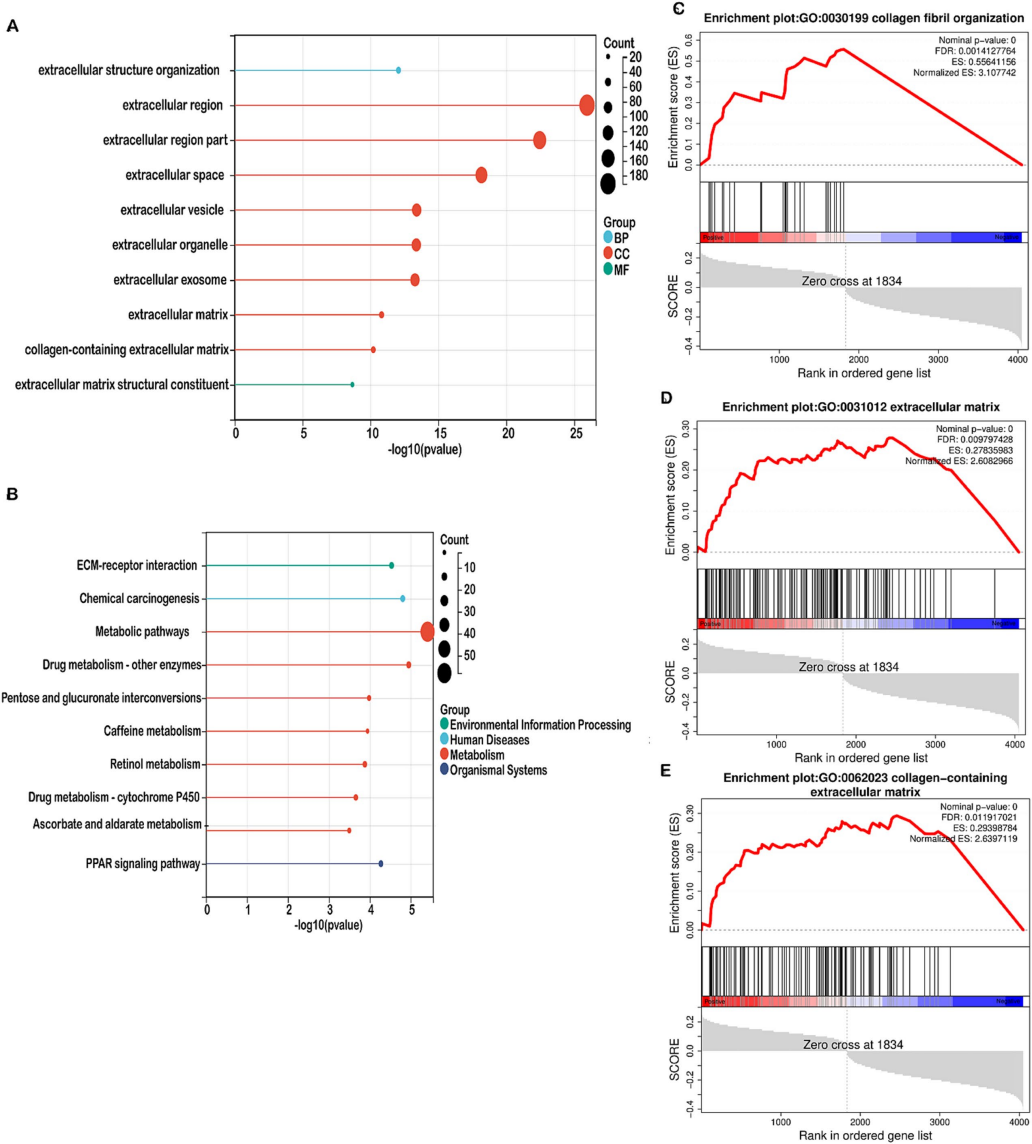
Enrichment analysis in the high-risk and low-risk groups. **(A)** Bubble map showing the 10 significant GO pathways, with bands of different colors representing biological process (BP), cellular component (CC), and molecular function (MF). The pathways were enriched by the genes listed on the left. **(B)** Bubble map illustrating the top 10 significant KEGG pathways, with bands of different colors representing each pathway. The pathways were enriched by the genes listed on the left. **(C–E)** GSEA identified different gene sets in the high-risk groups.

We performed functional enrichment analyses on differentially expressed genes (DEGs) within the high-risk and low-risk groups. Gene Ontology (GO) enrichment analysis indicated that DEGs linked to biological processes were primarily involved in extracellular structure organization (Figure 5A). Cellular component-related DEGs were predominantly associated with the extracellular region, extracellular space, and extracellular vesicle (Figure 5A). In terms of molecular functions, DEGs were enriched in extracellular matrix structural constituents (Figure 5A). The top 10 pathways identified by KEGG analysis included ECM-receptor interaction, chemical carcinogenesis, metabolic pathways, drug metabolism, pentose and glucuronate interconversions, caffeine metabolism, retinol metabolism, ascorbate and aldarate metabolism, and the PPAR signaling pathway (Figure 5B). Gene Set Enrichment Analysis (GSEA) revealed that mitochondrial metabolism-related risk scores in the high-risk group were significantly associated with collagen fibril organization, collagen-containing extracellular matrix, and the extracellular matrix (Figures 5C–E). GO and GSEA analyses were performed. Both the GO enrichment analysis and GSEA emphasized the role of the extracellular matrix, including terms like extracellular matrix organization, extracellular space, and extracellular vesicles. Moreover, identified by KEGG analysis, ECM-Receptor Interaction Pathway is closely linked to the TME. Interactions between cells and the ECM are crucial for signaling within the tumor microenvironment, impacting cell adhesion, migration, and invasion. Given that the tumor microenvironment consists largely of the extracellular matrix and signaling molecules that affect tumor behavior, our functional enrichment analyses highlight several key pathways (ECM organization, receptor interactions, collagen-related processes) that are integral to the TME. Therefore, this analysis provides evidence that TME-associated pathways were indeed enriched.

### Mitochondrial metabolism-related risk score and TME signatures in COADREAD

**Figure 6:**
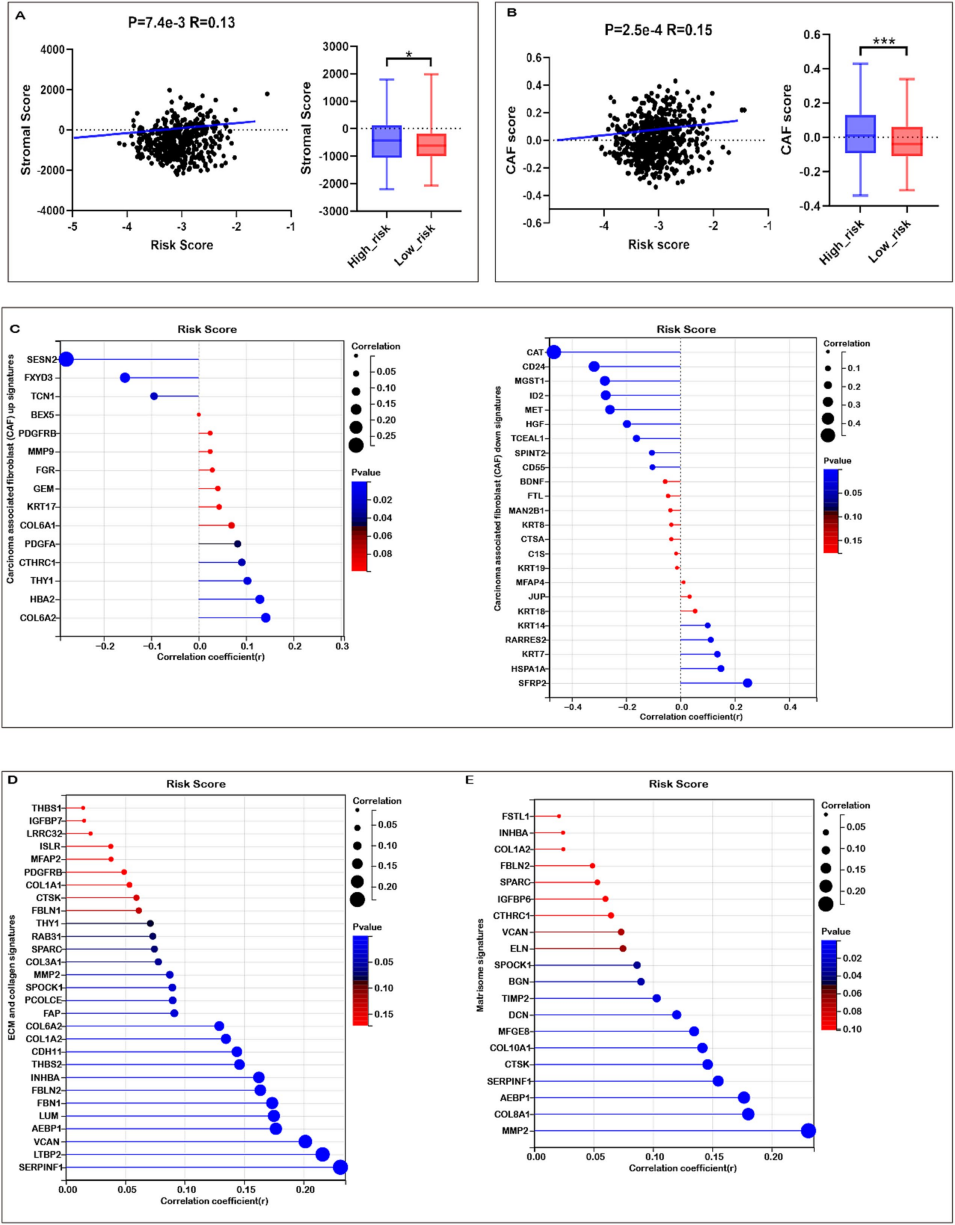
Association of risk score with tumor microenvironment (TME) Signatures in COADREAD. **(A)** Association between stromal score and risk score, and its distribution in the low- and high-risk groups. **(B)** Association between carcinoma-associated fibroblast (CAF) score and risk score, and its distribution in the low- and high-risk groups. **(C)** Correlation analysis of risk score with the expression of carcinoma-associated fibroblast (CAF) up- and down-signatures. **(D)** Correlation analysis of risk score with the expression of ECM and collagen signatures. **(E)** Correlation analysis of risk score with the expression of matrisome signatures. *p*-values are indicated as: ***p < 0.001, **p < 0.01, *p < 0.05.

Given the enrichment of TME-associated signaling pathways identified through functional analyses, we investigated the correlation between the risk score and TME signatures. As illustrated in Figure 6A, a strong positive correlation exists between the risk score and stromal score in COADREAD, with elevated stromal scores observed in the high-risk group relative to the low-risk group. Additionally, our analysis revealed a significant positive correlation between the risk score and the cancer-associated fibroblast (CAF) score (Figure 6B). Elevated CAF scores were notably present in the high-risk group compared to the low-risk group, highlighting the role of CAFs in tumor progression and prognosis in CRC. Our findings revealed significant positive correlations between the risk score and the expression of multiple CAF signatures (Figure 6C), as well as ECM-collagen and matrisome signatures (Figures 6D,E). These associations highlight the role of these signatures in CRC prognosis and potential therapeutic targeting. These observations suggest a close linkage between mitochondrial metabolism gene-related risk scores and TME signatures in COADREAD.

**Figure 7:**
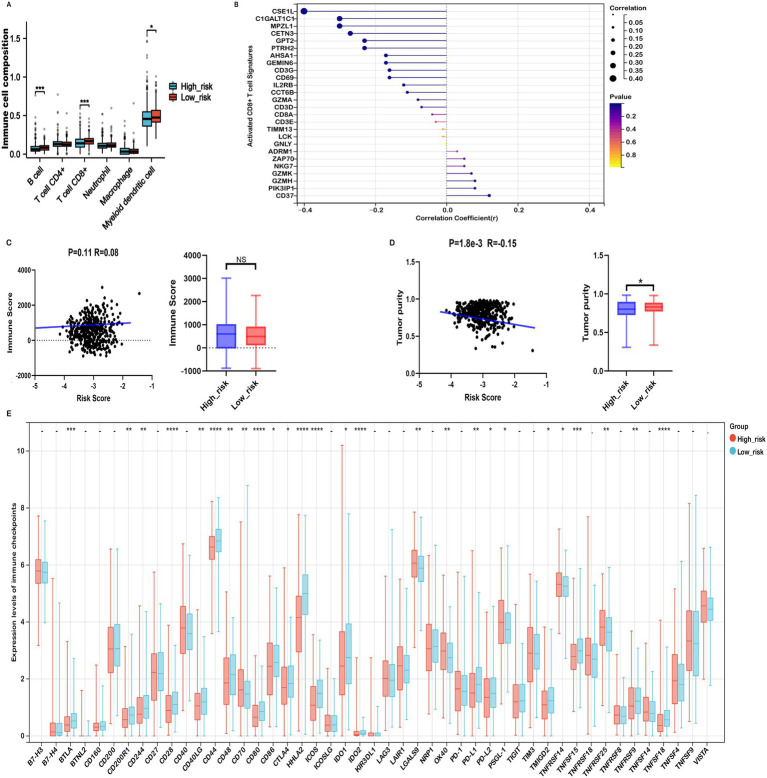
Immune profiles comparison between low- and high-risk groups in the TCGA-COADREAD dataset. **(A)** EPIC analysis. **(B)** Correlation between risk score and expressions of activated CD8+ T cell signatures. **(C)** Correlation between risk score and immune score, and its distribution in the low- and high-risk groups. **(D)** Correlation between risk score and tumor purity, and its distribution in the low- and high-risk groups. **(E)** Variation in immune checkpoint expression. *p*-values are indicated as follows: ***p < 0.001, **p < 0.01, *p < 0.05, ns (not significant).

The tumor immune microenvironment (TIME) critically influences therapeutic efficacy and prognosis in malignant tumors. Investigating the association between risk scores and immune cell infiltration in COADREAD is important for optimizing treatment strategies. EPIC analysis revealed that high-risk patients were associated with significantly reduced levels of B cells and CD8+ T cells (Figure 7A), consistent with a potentially compromised immune response. Furthermore, the risk score demonstrated a negative correlation with activated CD8+ T cell signature expression (Figure 7B), suggesting a potential decrease in anti-tumor immunity among high-risk individuals. This finding was supported by a parallel negative correlation with central memory CD8+ T cell signatures (Supplementary Figure S7), indicating reduced abundance of these key immune cells in high-risk patients.

ESTIMATE analysis showed that high-risk patients tended to have higher immune scores than their low-risk counterparts, although the difference did not reach statistical significance (Figure 7C). Further immune infiltration analysis using quanTIseq revealed that macrophage M1, neutrophils, NK cells, and CD8⁺ T cells were significantly more abundant in the low-risk group (Supplementary Figure S8). Moreover, macrophage M1 and neutrophils—cell types commonly associated with anti-tumor functions—were negatively correlated with the risk score (Supplementary Figures S9A,C), while immunosuppressive macrophage M2 cells were more prevalent in the high-risk group and positively correlated with risk score (Supplementary Figure S9B). These associations suggest that differences in immune cell composition may contribute to the observed prognostic differences across risk groups, although causal relationships cannot be established.

In addition, ESTIMATE analysis indicated that high-risk patients exhibited elevated stromal scores and significantly lower tumor purity compared to the low-risk group (Figures 6A, 7D). The risk score showed a positive correlation with matrisome and cancer-associated fibroblast (CAF) signatures, and a negative correlation with activated CD8⁺ T cell signatures. Notably, activated CD8⁺ T cell signatures were inversely associated with ECM, collagen, matrisome, and CAF signatures (Supplementary Figure S10), suggesting that a fibroblast- and ECM-rich environment may be unfavorable to CD8⁺ T cell activation.

Collectively, these findings reveal a complex association between the prognostic risk score and components of the TME, including immune and stromal features. While our data indicate that high-risk patients may harbor a more immunosuppressive TME, further mechanistic studies are required to confirm causality and elucidate the underlying biological interactions.

### Mitochondrial metabolism-related risk score was associated immune checkpoint inhibitors and immunotherapy responses in COADREAD

Considering the potential of Immune checkpoint inhibitors (ICIs) as a treatment for cancer, we examined the relationship between immune checkpoints and risk stratification. Our findings revealed that 25 immune checkpoints were significantly altered in the high-risk group (Figure 7E). Moreover, the risk score showed a significant positive correlation with the expression levels of seven immune checkpoints, including CD40, OX40, TNFRSF9, TNFRSF14, TNFSF18, TNFRSF25, CD70, PSGL-1, TNFRSF8, and TNFSF14 (r > 0.15, Supplementary Figure S11). In the context of advanced COADREAD treatment, inhibitors targeting PD-1 and CTLA-4 are currently areas of intense research interest. As illustrated in Figure 7E, the expressions of PD-L1 and CTLA-4 were significantly lower in the high-risk group. Consistently, the risk score exhibited a significant negative correlation with the expressions of PD-L1, PD-L2, and CTLA-4 (Supplementary Figure S11).

**Figure 8:**
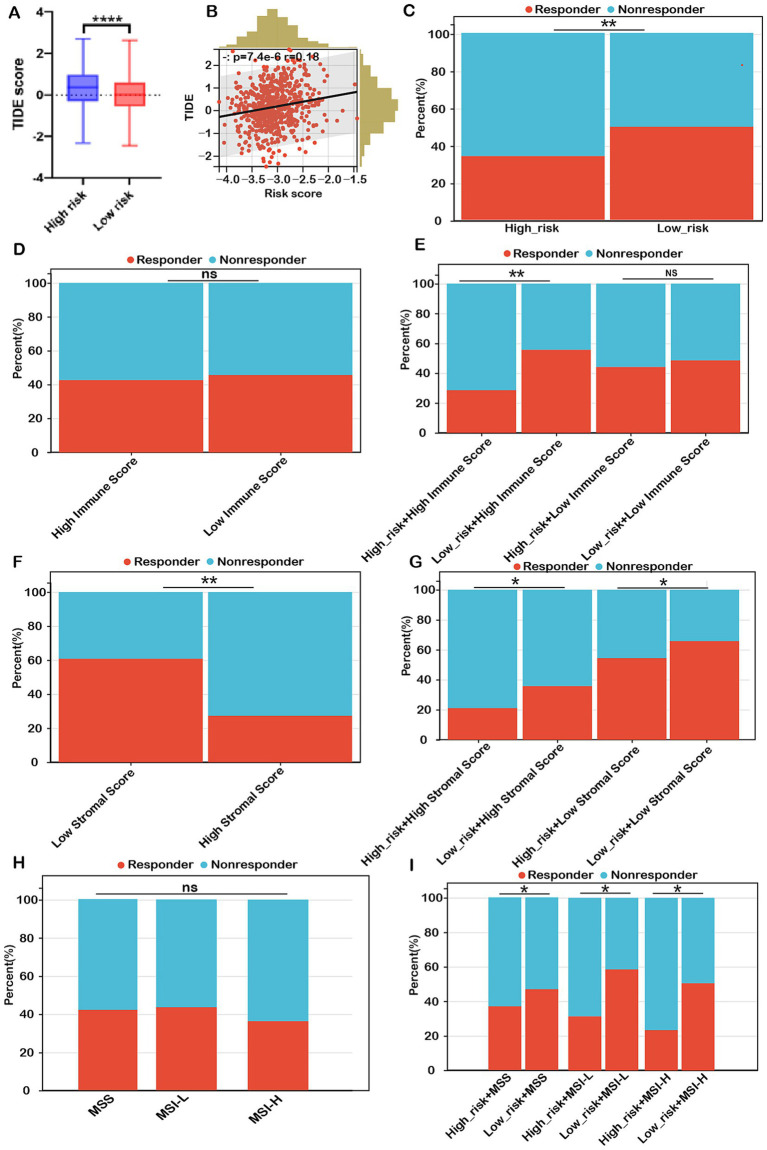
Risk score as a potential biomarker for predicting benefits from immune therapies in COADREAD. **(A)** Comparison of TIDE scores between low- and high-risk groups. **(B)** Correlation analysis between risk score and TIDE score. **(C)** Predicted proportion of immunotherapy responders in low- and high-risk groups within the TCGA-COADREAD cohort. **(D)** Predicted response rates to immunotherapy in patients with low and high immune scores (stratified by median cutoff), based on TIDE analysis. **(E)** TIDE-predicted response rates in four subgroups stratified by both risk score and immune score. **(F)** Predicted response rates in low- and high-stromal score groups (stratified by median cutoff). **(G)** TIDE-estimated immunotherapy responsiveness in four groups stratified by risk score and stromal score. **(H)** Predicted proportion of responders across different microsatellite statuses (MSS, MSI-L, and MSI-H). **(I)** TIDE-predicted immunotherapy response across six subgroups categorized by both risk score and microsatellite status. MSS, microsatellite stability (*n*=403); MSI-L, microsatellite instability-low (*n*=93); MSI-H, microsatellite instability-high (*n*=82). *p*-values are indicated as follows: ns (not significant); ***p < 0.001, **p < 0.01, *p < 0.05.

To corroborate these findings, we employed the TIDE algorithm to forecast immunotherapy responses in both low- and high-risk patient cohorts. The results indicated that the high-risk group exhibited a markedly higher TIDE score compared to the low-risk group (Figure 8A). Our analysis revealed a significant positive correlation between the risk score and the TIDE score (Figure 8B). The response rate to immunotherapy in the high-risk group (36.3%) was substantially lower than that in the low-risk group (51.7%) (Figure 8C). These findings suggest that patients in the low-risk group, with a lower TIDE score, are more likely to benefit from immune checkpoint inhibitor therapy and experience better survival outcomes following immunotherapy.

Conversely, immunotherapy response rates in the high-immune group (42.4%) were similar to those in the low-immune group (45.3%) (Figure 8D). Within the low-immune subgroup, immunotherapy response rates were 47.1% for the low-risk subgroup and 43.3% for the high-risk subgroup, similar to the overall low-immune group (45.3%). This indicates that combining the risk score with the immune score did not improve the prediction of immunotherapy response over the immune score alone for COADREAD patients with low immune scores. However, in the high-immune score subgroup, the immunotherapy response rate was significantly higher in the low-risk + high-immune group (56.7%) compared to the high-immune group (42.4%), while the high-risk + high-immune group had a notably lower response rate (30.3%) than the high-immune group (42.4%) (Figure 8E). This strongly suggests that the combination of risk score and immune score provides a more accurate prediction of immunotherapy response in COADREAD patients with high immune scores (Figure 8E). Overall, integrating risk score and immune score proves to be a robust predictor for immunotherapy responses in COADREAD.

As depicted in Figure 8F, the immunotherapy response rate in the low-stromal subgroup (60.1%) was significantly higher than in the high-stromal subgroup (27.6%). Notably, within the low-stromal score subgroup, the low-risk group had a considerably higher immunotherapy response rate (64.8%) compared to the high-risk group (54.5%) (Figure 8G). Additionally, in the high-stromal score subgroup, the low-risk group demonstrated a significantly greater immunotherapy response rate (35.1%) than the high-risk group (21.6%) (Figure 8G). These results indicate that combining risk score with stromal score enhances the prediction of immunotherapy responses in COADREAD.

The microsatellite instability-high (MSI-H) phenotype characterizes a distinct tumor type with a significant potential for immunotherapy. However, our analysis showed no significant difference in immunotherapy response rates between the MSI-H subgroup and the MSS and MSI-L subgroups (all *p* > 0.05, Figure 8H). Notably, within the MSS subgroup, the low-risk group exhibited a higher response rate (48.3%) compared to the high-risk group (37.3%) (Figure 8I). Similarly, the MSI-L subgroup demonstrated a higher response rate in the low-risk group (59.1%) versus the high-risk group (31.8%). In the MSI-H subgroup, the low-risk group had a response rate of 50.1%, significantly higher than the 23.9% observed in the high-risk group (Figure 8I). These results suggest that combining risk score with MSI status can reliably predict immunotherapy response in COADREAD patients (Figure 8I). Graph summarization of immunotherapy responses in CRC was depicted in Supplementary Figure S12. These results suggest that combining risk scores with various biomarkers, including immune scores, stromal scores, and microsatellite instability (MSI), has significantly enhanced our ability to predict immunotherapy responses in colorectal cancer (CRC).

### Mutation status of CRC patients in high-risk and low-risk groups

**Figure 9:**
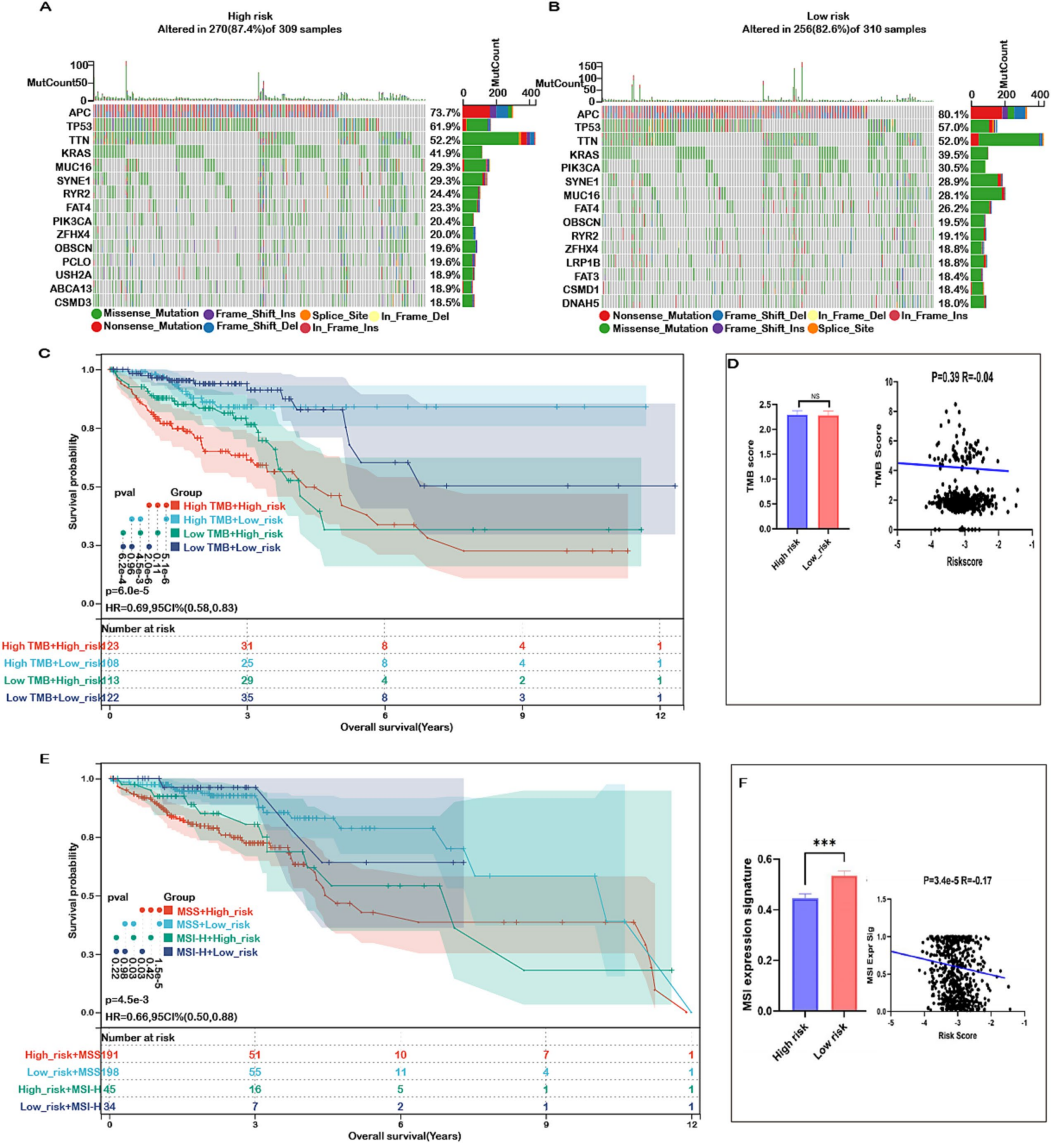
Mutation status in high- and low-risk groups in COADREAD. **(A)** Top 15 genes according to mutation frequency in high-risk groups. **(B)** Top 15 genes according to mutation frequency in low-risk groups. **(C)** Kaplan–Meier curves of OS of patients in high- and low-TMB groups combined with risk score in the TCGA-COADREAD cohort. **(D)** TMB score distribution in the low- and high-risk groups. Correlation between risk score and TMB in COADREAD. **(E)** Kaplan–Meier curves of OS of patients in MSS and MSI-H groups combined with risk score in the TCGA-COADREAD cohort. **(F)** MSI expression signature distribution in the low- and high-risk groups. Correlation between risk score and MSI expression signature in COADREAD. *p*-values are indicated as follows: ns (not significant); *p < 0.05; ***p < 0.001.

Mutations accumulate over a person’s lifetime, contributing to cancer development. Advances in genome sequencing technology have significantly enhanced our understanding of the somatic mutations driving cancer, shedding light on mutational processes and identifying key oncogenes. Therefore, our study analyzed the mutation landscape of COADREAD, stratifying patients into high-risk and low-risk groups based on their risk scores. Among the most frequently mutated genes in both groups were APC, TP53, TTN, KRAS, MUC16, SYNE1, RYR2, FAT4, PIK3CA, ZFHX4, OBSCN, and CSMD3 (Figures 9A,B). The survival analysis revealed no significant difference in OS between the high-TMB and low-TMB groups (Supplementary Figure S13A). However, patients in the low-risk group exhibited better overall survival (OS) compared to those in the high-risk group across both high and low TMB subgroups (Figure 9C). Additionally, comprehensive analysis revealed no significant difference in tumor mutational burden (TMB) between the high-risk and low-risk groups (Figure 9D). Furthermore, there was no meaningful correlation between risk score and TMB (Figure 9D), indicating that TMB alone may not be sufficient to stratify risk in COADREAD. This suggests that the integration of risk score with TMB could provide a more nuanced prognostic biomarker, potentially guiding therapeutic decisions and improving patient outcomes.

These findings underscore the importance of integrating multiple biomarkers to improve prognostic accuracy in colorectal cancer. Microsatellite instability (MSI), a key immunotherapy marker, was significantly lower in the high-risk group and showed a negative correlation with the risk score (Figure 9F). While patients with high MSI (MSI-H) generally exhibited better overall survival than those with low MSI (MSS), the difference was not statistically significant (Supplementary Figure S13B). Importantly, within both MSI subgroups, patients in the low-risk category consistently demonstrated superior survival compared to those in the high-risk group (Figure 9E). This indicates that combining risk score with MSI status enhances prognostic prediction and may better inform treatment strategies for COADREAD patients.

### Mitochondrial metabolism genes-related risk score and chemotherapy response

**Figure 10:**
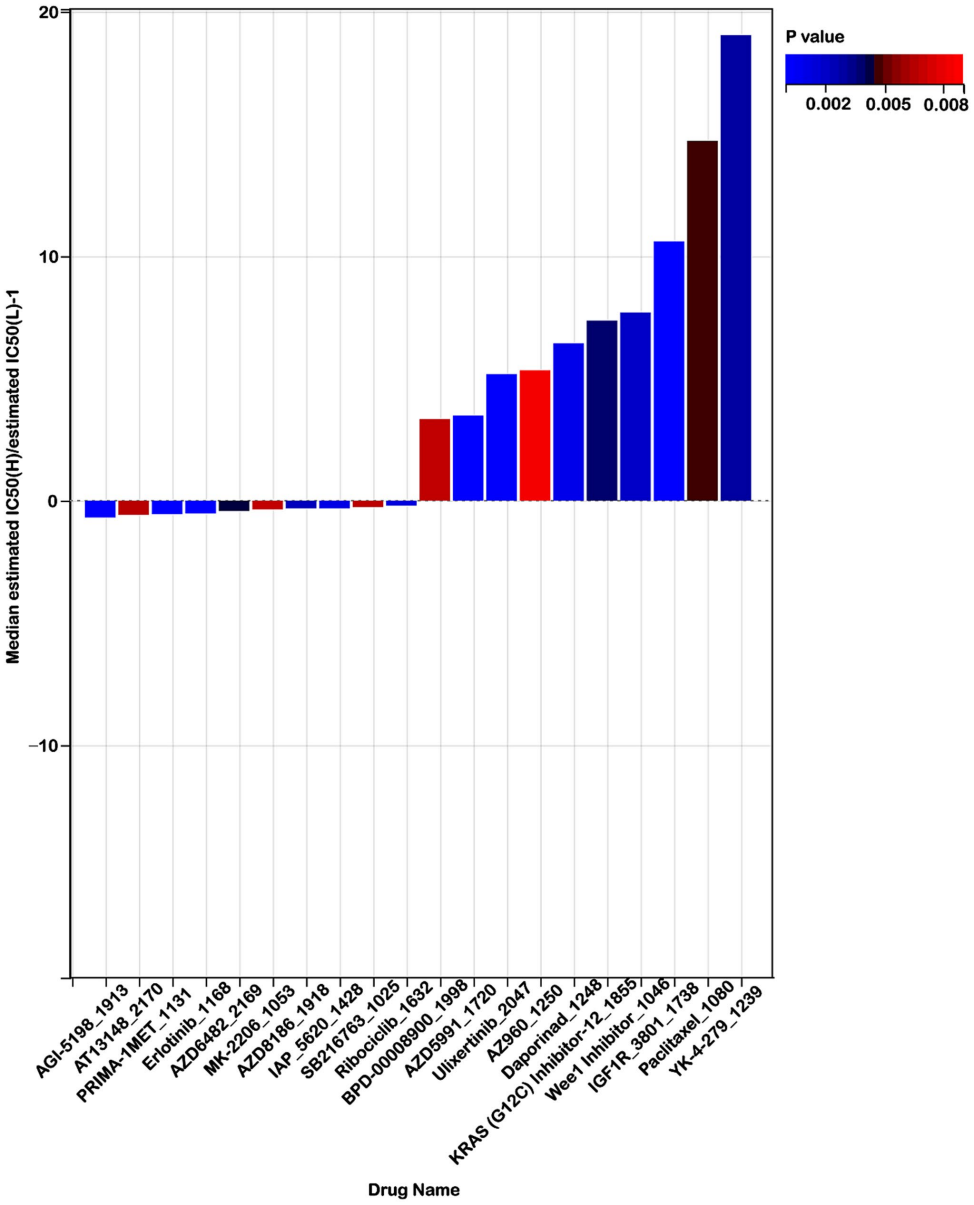
Risk score predicts drug therapeutic benefits in colon cancer. Proportion of normalized IC50 values of the top 10 sensitivity drugs in high-risk low-risk groups (*p* < 0.01).

To explore the effectiveness of risk score as an indicator for predicting the response to drugs, we estimated the IC50 values for 198 drugs in patients from the TCGA cohort. The top 10 sensitivity drugs in high-risk low-risk groups were shown in Figure 10. Our analysis indicated that individuals in the high-risk group might exhibit greater sensitivity to drugs such as AGI-5198_1913, AT13148_2170, and PRIMA-1MET-1131 (Figure 10). Conversely, those in the low-risk group might respond more favorably to treatments like YK-4-279_1239, IGF1R_3801_1738, and Paclitaxel_1080 (Figure 10). These findings offer valuable insights for guiding clinical treatment strategies.

### Verification of the expression levels of mitochondrial metabolism-related core genes in CRC samples

In our previous analysis (Supplementary Figure S5), multifactorial Cox regression identified TMEM86B, TNFAIP8L3, and HDC as independent prognostic factors for colorectal cancer (CRC) (*p* < 0.05) among the 15 mitochondrial metabolism-related genes. To further evaluate their expression, we analyzed data from the TCGA (Figure 2F) and GEO (Supplementary Figure S3) databases. TMEM86B was found to be upregulated in CRC tissues compared to normal tissues, while TNFAIP8L3 and HDC showed significantly lower expression in tumors. To validate these findings, qRT-PCR was performed on six paired CRC tumor and adjacent normal tissue samples. As shown in Supplementary Figure S14A, TMEM86B expression was significantly higher in tumor tissues (*p* < 0.05), whereas TNFAIP8L3 and HDC levels were significantly lower in tumors compared to normal tissues (*p* < 0.05, Supplementary Figures S14B,C). These results corroborate our hypothesis and provide strong support for including these three genes in our prognostic model (Supplementary Figure S5).

### Knockdown of TMEM86B inhibited CRC cell proliferation and migration *in vitro* and *in vivo*

**Figure 11:**
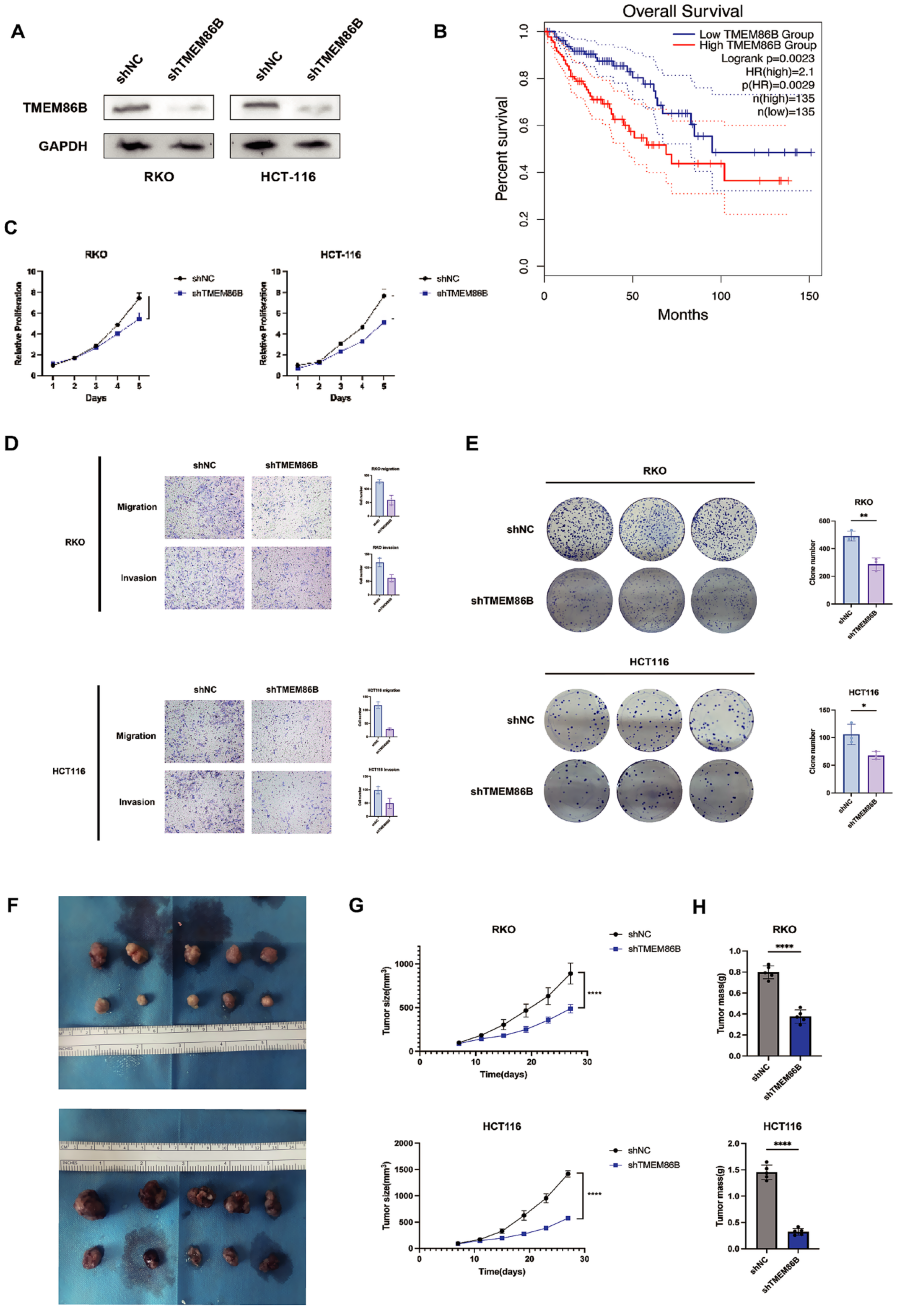
Knockdown of TMEM86B inhibited CRC cells proliferation and migration in vitro and *in vivo*. **(A)** Western blot analysis of TMEM86B knock-down stably transfected cell lines. **(B)** Survival curve of TMEM86B in TCGA-COAD cohort in GEPIA2. **(C,E)** Clone formation and CCK8 assay of CRC cell lines with TMEM86B perturbation. 3 independent experiments were conducted, and data were shown with Mean ± SD (two-tailed t-test, **, *p* < 0.01, ***, *p* < 0.001). **(D)** Representative images of migration and invasion after TMEM86B was silenced in RKO and HCT116. **(F)** Representative images of resected subcutaneous tumors. **(G)** Subcutaneous tumor dimensions were recorded using calipers at every 4 days. And tumor volume was calculated by formula: Length x Width2/2 (mean ± SD, *n* = 5 for each group, one-way ANOVA, ***, *p* < 0.001). **(H)** Tumor weight was recorded at time of harvest and plotted according to treatment group (Mean ± SD, two-tailed t-test, *, *p* < 0.05).

Of the 15 prognostic genes identified, TMEM86B was prioritized for experimental validation based on its strong prognostic relevance, as indicated by a high hazard ratio in Cox regression and consistent overexpression in tumor samples. Given that its functional role in CRC remains largely unexplored, particularly in the context of mitochondrial metabolism, we aimed to characterize its potential contribution to colorectal tumorigenesis through subsequent *in vitro* and *in vivo* analyses. To evaluate the clinical significance of TMEM86B in colorectal cancer, we analyzed data from the GEPIA2 database, which showed that elevated TMEM86B expression was significantly associated with poorer prognosis in colon cancer patients (Figure 11B). This finding suggests that TMEM86B may play a role in promoting the aggressive behavior of CRC.

To further explore the biological function of TMEM86B in CRC, we generated TMEM86B knockdown cell lines by lentiviral transduction in the RKO and HCT116 CRC cell lines (RKO/shTMEM86B, HCT116/shTMEM86B) (Figure 11A). Functional assays demonstrated that silencing TMEM86B effectively inhibited CRC cell viability, as evidenced by reduced cell proliferation and clonogenicity in both the cell proliferation assays and colony formation assays (Figures 11C,E). These results indicate that TMEM86B is involved in promoting CRC cell growth.

Furthermore, we assessed the migratory and invasive capabilities of CRC cells after TMEM86B knockdown. A marked decrease in the migration and invasion potential of the RKO/shTMEM86B and HCT116/shTMEM86B cells was observed, indicating that TMEM86B plays a critical role in promoting CRC cell motility (Figure 11D).

In addition to the in vitro findings, we examined the impact of TMEM86B knockdown on tumor growth in vivo. Subcutaneous xenograft models were established by transplanting RKO/shTMEM86B and HCT116/shTMEM86B cells into nude mice (Figure 11F). Tumor growth was monitored by measuring tumor volume and weight. As shown in Figures 11G and 11H, both the average tumor volume and weight were significantly decreased in the TMEM86B knockdown groups compared to their respective controls (RKO/shNC vs. RKO/shTMEM86B; HCT116/shNC vs. HCT116/shTMEM86B), indicating that TMEM86B down-regulation suppresses tumor growth in vivo.

In conclusion, our findings provide compelling evidence that TMEM86B contributes to the proliferation, migration, and invasion of CRC cells, both in vitro and in vivo. The high expression of TMEM86B correlates with poor prognosis in CRC patients, suggesting its potential as a novel therapeutic target in CRC treatment.

## Discussion

Colorectal cancer (CRC) is a leading cause of cancer-related morbidity and mortality worldwide, ranking third in incidence and second in death rates globally (25). The prognosis for CRC patients is significantly influenced by the stage at which the tumor is detected, with a general 5-year overall survival rate of about 65% (26). Recent advances in cancer biology have revealed that mitochondrial energy metabolism is reprogrammed in malignant cells to support their rapid growth, invasion, and metastasis (27). Given the central role of mitochondria in tumorigenesis, they present promising therapeutic targets (28). However, there remains a paucity of studies focused on the prognostic significance of mitochondrial metabolism-related genes in CRC, particularly in constructing predictive models. This study aims to fill this gap by identifying mitochondrial metabolism-related genes that could serve as prognostic biomarkers, facilitating early intervention and personalized therapy for high-risk patients.

Currently, many biomarkers were applied for prognostic prediction of CRC, such as GPSM2, TNFAIP8L3, HDC and NDUFA4L2, but most of them are studied for a single biomarker (29–32). Increasing evidences indicated that prognostic model constructed by multi-genes as a prognostic index was more comprehensive and effective than single gene in kinds of malignancies. For instance, Q. Li et al. constructed a CRC prognosis model based on Treg-related genes, associated with Treg infiltration (33). A Huang et al. constructed an immune-related prognostic model hypoxia- and lactate metabolism-related molecular subtyping and prognostic signature for colorectal cancer (34). As the dysfunction of mitochondrial metabolism have been associated with cancer, we constructed a CRC prognostic model based on mitochondrial metabolism-related genes that could be used to predict the prognosis and efficacy of immunotherapy in patients with CRC.

In this study, a total of 582 mitochondrial metabolism-related genes were identified through the MSigDB and TCGA databases. Subsequent analyses, including univariate Cox regression and least absolute shrinkage and selection operator (LASSO) regression, narrowed the candidate list to 15 key genes. Among these, five genes (HSD3B7, NDUFA4L2, TMEM86B, FABP4, and TNFAIP8L3) were associated with increased risk, whereas ten genes (ORC1, GPSM2, CHDH, LARS2, HMGCL, GDE1, ACOX1, ARV1, HDC, and GSRL3) were linked to reduced risk. Notably, many of these genes have previously been implicated in colorectal cancer (CRC) progression, highlighting their potential utility as prognostic biomarkers.

To validate the predictive model, we performed internal and external analyses. Internal validation through ROC curve analysis revealed excellent diagnostic capability, with an area under the curve (AUC) of 0.74, demonstrating strong sensitivity and specificity. Compared to existing prognostic models, our mitochondrial metabolism-related signature demonstrated superior predictive accuracy, achieving a 23.3% higher AUC than the RNA-binding protein model (0.74 vs. 0.60), and outperforming both the mitophagy (0.74 vs. 0.64) and autophagy models (0.74 vs. 0.66) (35–37). Notably, it also surpassed several other CRC models based on metabolic and immune-related genes, including those associated with nucleotide metabolism, immune profiles, immunogenic cell death, CD4⁺ T cells, and metastasis-immune interactions (38–44), consistently achieving the highest AUC (0.74). These results highlight the unique prognostic value of mitochondrial metabolism genes, which may more accurately reflect tumor bioenergetics and microenvironmental interactions, offering a more precise and clinically applicable tool for CRC risk stratification. Kaplan–Meier survival analysis showed significantly poorer outcomes for high-risk patients. External validation using GEO datasets confirmed these results, with low-risk patients having better overall survival. These findings highlight the reliability of mitochondrial metabolism-related genes in CRC prognosis.

TMEM86B, TNFAIP8L3, and HDC were identified as independent prognostic markers, offering new insights into the role of mitochondrial dysfunction in CRC. TMEM86B, involved in plasmalogen metabolism and mitochondrial function, was significantly overexpressed in CRC and associated with poor prognosis, suggesting a potential oncogenic role through disrupted lipid metabolism and mitochondrial imbalance (45, 46).

Similarly, TNFAIP8L3, known to mediate mitochondrial stress and tumor progression in other cancers (47, 48), also showed elevated expression in CRC patients with unfavorable outcomes, possibly contributing to immune evasion. In contrast, HDC was downregulated in CRC and linked to worse prognosis, differing from its oncogenic role in glioblastoma (49), suggesting a context-dependent function. Together, these findings highlight the potential involvement of these genes in CRC progression via mitochondrial pathways, though further mechanistic studies are needed.

TMEM86B was selected for further investigation due to its strong prognostic significance, as evidenced by high positive coefficients and hazard ratios in both univariate and multivariate Cox regression analyses, suggesting it may serve as an independent prognostic factor. Moreover, TMEM86B was consistently upregulated in tumor tissues compared to normal controls across multiple datasets. Considering the limited functional characterization of TMEM86B in colorectal cancer to date, it represents a promising candidate for uncovering novel oncogenic mechanisms. In this study, we examined its potential role in colorectal cancer progression and provided robust evidence that TMEM86B facilitates malignant phenotypes, underscoring its contribution to tumor development.

In conclusion, our findings provide compelling evidence that TMEM86B contributes to CRC progression by enhancing cell proliferation, migration, and invasion. Its high expression correlates with poor prognosis, positioning it as a promising prognostic biomarker and potential therapeutic target in CRC. Future studies focusing on the molecular mechanisms of TMEM86B could provide valuable insights into the development of targeted therapies for CRC treatment.

Although TMEM86B has been identified as a potential contributor to CRC progression, its underlying molecular mechanisms remain poorly defined. This represents a limitation of the current study and highlights the need for further investigation into the specific signaling pathways and metabolic alterations regulated by TMEM86B. Given its predicted mitochondrial membrane localization and involvement in lipid metabolism, TMEM86B may play a role in mitochondrial lipid remodeling, membrane integrity, or metabolic reprogramming—processes that are critical for tumor development and invasion. To elucidate these mechanisms, future studies employing transcriptomic profiling, functional rescue experiments, and metabolic flux analysis are warranted.

Further analysis of the differentially expressed genes (DEGs) between high- and low-risk groups revealed a significant enrichment of pathways related to extracellular matrix (ECM) processes, particularly ECM organization. This observation aligns with previous findings, which emphasize that ECM accumulation is a hallmark of aggressive tumor behavior and is commonly associated with poor prognosis in various cancer types (50). Within the TME, fibroblasts undergo transformation into cancer-associated fibroblasts (CAFs), which are highly prevalent in both primary and metastatic tumors. CAFs are known for their remarkable plasticity and resilience, and they exert significant influence on cancer progression through interactions with other components of the TME (51, 52, 69, 70). The matrisome, a collective term referring to genes encoding core ECM proteins and structural components, is fundamental to understanding cancer biology (53). Yuzhalin et al. identified a common nine-gene matrisome signature that is overexpressed in several cancers, including breast, gastric, lung, ovarian, and colorectal cancers (54). Consistent with these findings, our analysis identified a strong positive correlation between the risk score and the expression of CAF, ECM, and matrisome signatures. In addition, the risk score was positively correlated with the stromal score and negatively correlated with tumor purity, suggesting that a higher stromal content tends to be associated with the high-risk group in CRC. While these associations align with previous studies highlighting the prognostic relevance of stromal components in CRC, they do not imply a direct causal relationship. Further investigation is needed to determine whether stromal infiltration plays a mechanistic role in shaping prognosis or reflects other underlying tumor characteristics.

Immune cells are integral to the TME, contributing significantly to both tumor progression and responses to treatment. Recent studies have shown that distinct TME phenotypes correlate with different immunotherapeutic responses and clinical outcomes (55, 56). A key advantage of immunotherapy is its potential to induce memory CD8+ T cells, providing durable protection against tumor metastasis and recurrence (57). Emerging evidence suggests that these TME phenotypes are also linked to differential survival rates and variable responses to immunotherapy (58, 59). Considering the pivotal role of immune cells in the TME and their influence on therapeutic efficacy, we explored the variations in immune cell composition between high- and low-risk groups.

Among the immune components within the TME, our results demonstrated that M0 and M2 macrophages were significantly more abundant in the high-risk group. This pattern indicates that the high-risk group tends to be associated with a more immunosuppressive microenvironment, characterized by elevated levels of M0 and M2 macrophages. These macrophage subtypes have been implicated in tumor immune evasion through the suppression of CD8⁺ T cell responses in previous studies, although their precise functional role in our model remains to be clarified.

In contrast, the low-risk group exhibited higher levels of immune cells generally associated with anti-tumor immunity, including B cells, CD8⁺ T cells, CD4⁺ T cells, M1 macrophages, neutrophils, and natural killer (NK) cells. This distinct immune profile may reflect a more active anti-tumor immune response in the low-risk group. However, as our study is observational in nature, these associations should not be interpreted as evidence of causality. Further experimental work is needed to elucidate whether these immune cell populations actively mediate prognostic differences or serve as correlates of broader tumor biological processes.

Monoclonal antibodies targeting immune checkpoint molecules have marked a significant advancement in cancer treatment (60). Our study showed that high-risk patients had a significantly lower response to immunotherapy than low-risk patients, consistent with their lower expression of PD-1, PD-L1, and CTLA-4. Integrating the risk score with immune or stromal scores improved the prediction of immunotherapy response in COADREAD, highlighting its value as a potential biomarker. Clinical evidence shows that anti-PD-1/PD-L1 therapies are effective in dMMR/MSI-H colorectal cancers but less so in pMMR/MSS cases, likely due to their immunologically ‘cold’ tumor microenvironment. This suggests that MSI status, through its association with neoantigen generation and immune activation, is a reliable predictor of response to PD-L1 therapy (61, 62). In our study, while MSI status was negatively correlated with the risk score (Figure 9F), it alone failed to significantly distinguish patient survival (Supplementary Figure S13B). In contrast, combining MSI with the risk score improved prognostic power. In both MSS and MSI-H subgroups, low-risk patients had better survival than high-risk ones (Figure 9E), and showed higher immunotherapy response rates across all MSI types, especially in MSI-H (50.1% vs. 23.9%, Figure 8I). These findings highlight the added prognostic and predictive value of our model beyond MSI alone. Our findings also suggest potential clinical applications of the prognostic risk score in guiding immunotherapy decisions for CRC patients. Given the observed associations between low-risk scores and a more immune-activated tumor microenvironment—characterized by higher infiltration of CD8⁺ T cells, NK cells, and M1 macrophages—these patients may be more likely to benefit from ICIs. In contrast, high-risk patients demonstrated features of an immunosuppressive TME, including increased M2 macrophages and stromal activation, which may impair immunotherapy efficacy. Thus, the risk model may serve as a tool for stratifying patients and selecting personalized treatment strategies. For high-risk individuals, combination therapies (e.g., ICIs plus CAF-targeted agents or anti-fibrotic treatments) could be explored to overcome immune resistance.

Our analysis showed no significant difference in TMB levels between high- and low-risk groups, and no strong correlation between TMB and the risk score (Figure 9D). Survival also did not differ significantly between high- and low-TMB patients alone (Supplementary Figure S13A), suggesting TMB alone is insufficient for prognosis.

However, combining TMB with our risk score improved stratification. In both high- and low-TMB subgroups, low-risk patients had better survival than high-risk ones (Figure 9C). These findings suggest our model adds prognostic value beyond TMB and supports integrated approaches for more accurate risk assessment in colorectal cancer.

To further explore the translational potential of our drug sensitivity predictions, we examined existing evidence for the top-ranked compounds identified in our analysis. AGI-5198, a selective inhibitor of mutant IDH1 (R132H), has been shown to suppress tumor growth and induce differentiation in IDH1-mutant cancer cells, including colorectal cancer models, suggesting its promise in targeted therapy for specific molecular subtypes (63, 64). PRIMA-1MET (APR-246), a reactivator of mutant p53, has been reported to promote autophagy and apoptosis in CRC cells via the mTOR/AMPK-ULK1-Vps34 signaling pathway, highlighting its relevance in p53-mutant tumors (65). On the other hand, Paclitaxel, a microtubule-stabilizing agent, is already in clinical use and has shown benefit in certain CRC treatment settings, particularly in combination with other agents (66). YK-4-279, which blocks the EWS-FLI1 transcription factor, effectively kills p53-deficient colorectal cancer cells with the BRAFV600E mutation (like RKO) by stopping ETS1 increase and causing a parthanatos-like cell death involving overactive PARP1, mitochondrial damage, and AIF moving to the nucleus (67). IGF1R inhibitors have demonstrated antiproliferative effects in CRC models by disrupting oncogenic signaling pathways (68). These findings support the potential utility of our risk model not only in prognostic stratification but also in guiding individualized therapeutic strategies. By integrating genomic risk profiles with predicted drug sensitivities, our model may help identify patient subgroups that are more likely to benefit from specific targeted or chemotherapeutic agents, offering a path toward more personalized treatment in colorectal cancer.

This study offers several key advantages. The risk model, based on multiple mitochondrial metabolism-related genes, shows stronger prognostic value than single-gene methods and enables clear classification of COADREAD patients into high- and low-risk groups. It also predicts chemotherapy and immunotherapy responses, supporting personalized treatment. In addition, TMEM86B was identified as a key gene promoting colorectal cancer progression, suggesting its potential as a therapeutic target.

### Limitations

Despite the significant clinical implications of our findings, several limitations should be acknowledged. First, this study is retrospective in nature and relies primarily on publicly available databases, which may introduce inherent biases. Future validation in large-scale, prospective, multi-center clinical cohorts is necessary to confirm the stability and generalizability of the risk score model across diverse populations and clinical settings. Second, the oncogenic roles of the prognostic genes included in the model, as well as the underlying mechanisms by which these genes interact with mitochondrial metabolism in colorectal cancer, remain to be fully elucidated. Another limitation is the lack of histological analyses (e.g., H&E, Ki-67, cleaved caspase-3) in the *in vivo* experiments. Future studies will incorporate histological assessments to provide further mechanistic insights into tumor proliferation and apoptosis. Additionally, future studies should expand the analysis of mitochondrial-related genes using larger datasets to enhance the robustness and clinical applicability of our model.

## Conclusion

We developed a novel mitochondrial metabolism-related risk model for colorectal adenocarcinoma, which is closely associated with the tumor microenvironment and immune infiltration. Combining the risk score with stromal score, immune score, or MSS/MSI status improved prediction of immunotherapy response. Drug sensitivity analysis indicated distinct treatment responses between high- and low-risk groups. TMEM86B was identified as a potential oncogene, and its knockdown inhibited tumor growth *in vitro* and in vivo. Overall, this model may serve as a reliable prognostic biomarker and guide personalized therapy in COADREAD.

## Data Availability

The datasets presented in this study can be found in online repositories. The names of the repository/repositories and accession number(s) can be found in the article/Supplementary material.

## Footnotes

^1^ https://www.cancer.gov/tcga

^2^ http://xena.ucsc.edu

^3^ https://www.gsea-msigdb.org/gsea/msigdb

^4^ http://www.gsea-msigdb.org/gsea/index.jsp

^5^ https://portal.gdc.cancer.gov

^6^ https://www.cancerrxgene.org/

^7^ http://tide.dfci.harvard.edu

^8^ https://www.cbioportal.org/

## Glossary

COADREAD: Colon and Rectal Cancer
TME: Tumor microenvironment
CRC: Colon and Rectal Cancer
OS: Overall survival
ICB: Immune checkpoint blockade
TMB: Tumor mutation burden
GSEA: Gene set enrichment analyses
DEGs: Differentially expressed genes
K-M: Kaplan–Meier
C-index: Concordance index
HPA: The Human Protein Atlas
NCBI: National Center for Biotechnology Information
DCA: Decision curve analysis
GO: Gene Ontology
KEGG: Kyoto Encyclopedia of Genes and Genomes
TIICs: Tumor-infiltrating immune cells
IC50: 50% Inhibiting concentration
GDSC: Genomics of Drug Sensitivity in Cancer
TIDE: Tumor immune dysfunction and exclusion
ECM: Extracellular matrix
CAF: Carcinoma associated fibroblast
Tregs: Regulatory T cell
MSS: Microsatellite stability
MSI-L: Microsatellite instability-low
MSI-H: Microsatellite instability-high
PD-L1: Programmed death-ligand 1
TAMs: Tumor-associated macrophages
M2: Macrophage type 2
BP: Biological process
CC: Cellular component
MF: Molecular function
