# Supplementary material for: Constructing a novel mitochondrial metabolism-related genes signature to evaluate tumor immune microenvironment and predict survival of colorectal cancer
Source: Front Med (Lausanne). 2025 Jul 8;12:1618471. doi: 10.3389/fmed.2025.1618471 (PMC12279720; doi:10.3389/fmed.2025.1618471)
Supplement: Supplementary file 1 [file Data_Sheet_1.pdf]

Constructing a novel mitochondrial metabolism-related genes signature to evaluate tumor immune microenvironment and predict survival of colorectal cancer

Supplementary data

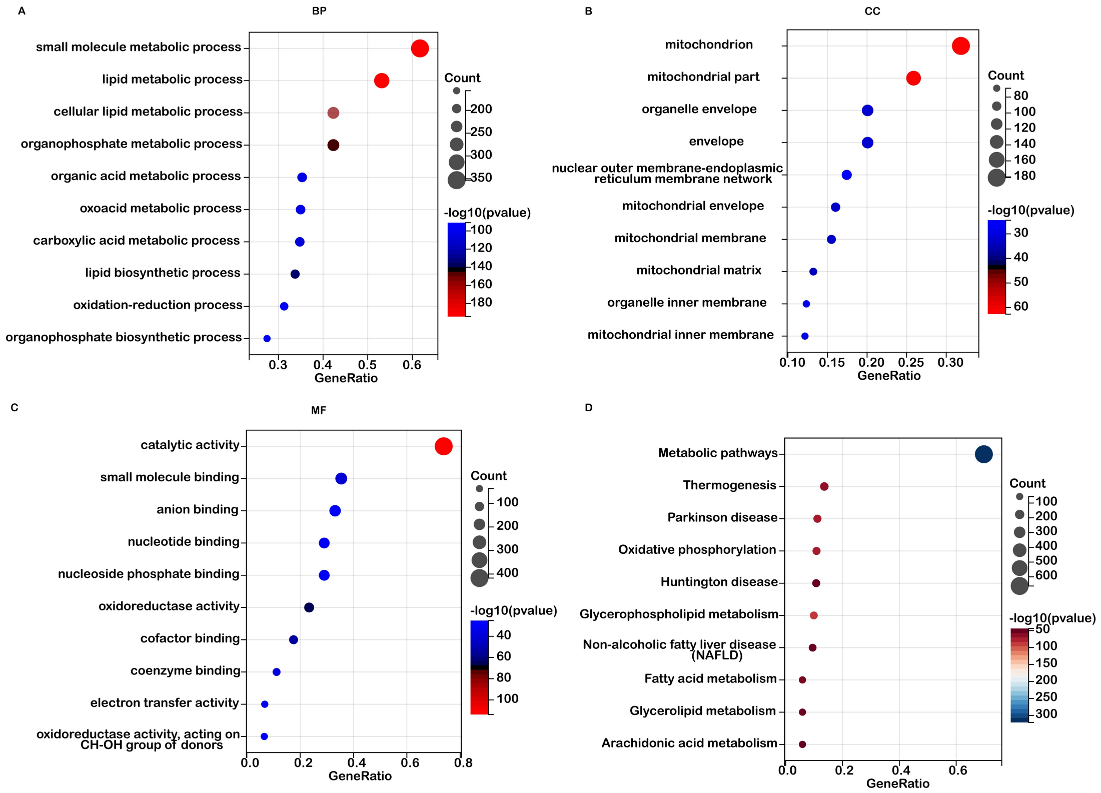

Figure S1. Identification of DEGs Related to Mitochondrion and Functional Enrichment Analysis in COADREAD.

GO analysis of 582 mitochondrial-related DEGs, including (A) biological process (BP), (B) cellular component (CC), and (C) molecular function (MF).(D) KEGG analysis of 582 mitochondrial-related DEGs.

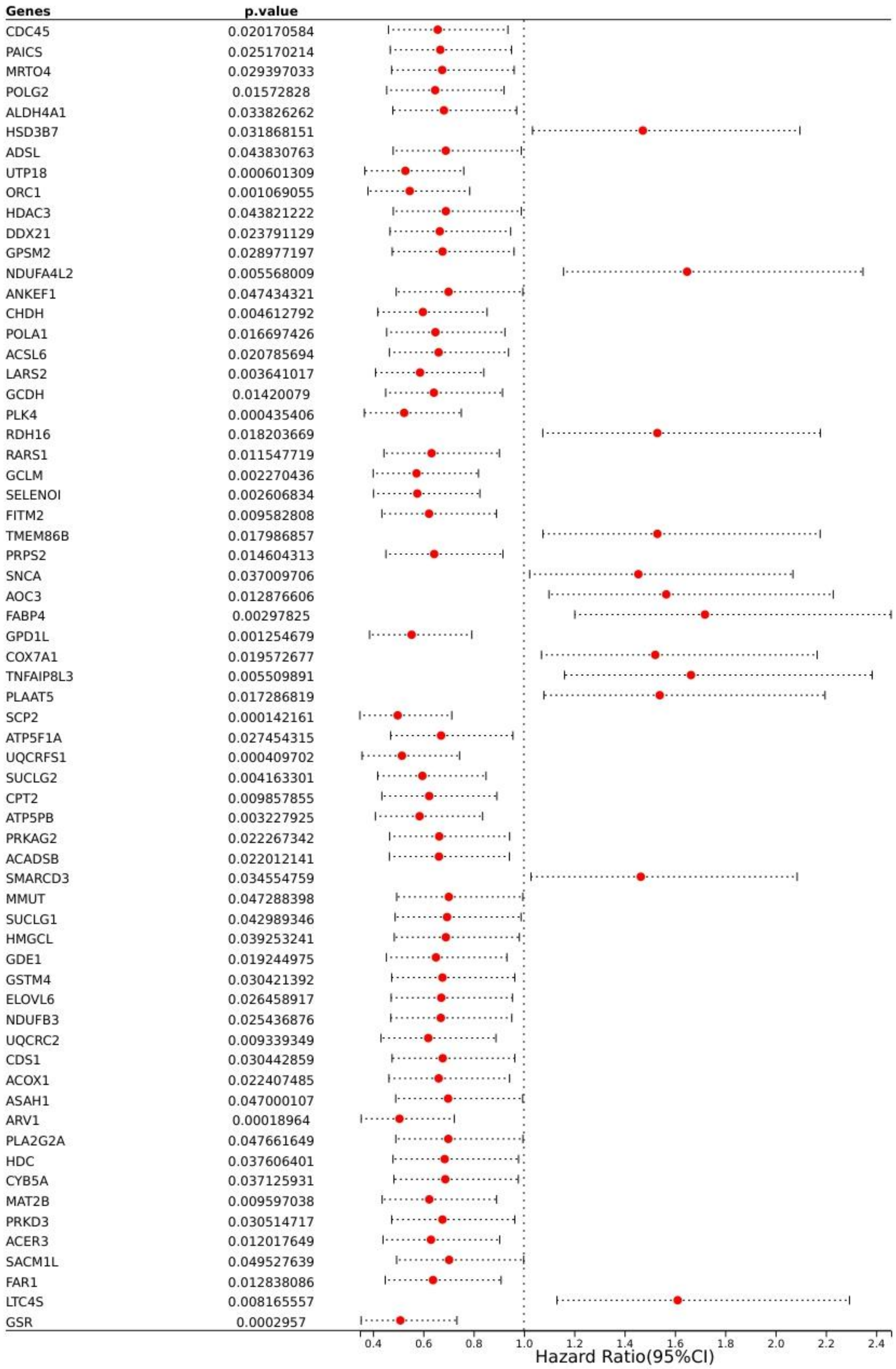

Figure S2. Identification of 65 Potential Prognostic DEGs for COADREAD through Univariate Cox Regression Analysis (P < 0.05)

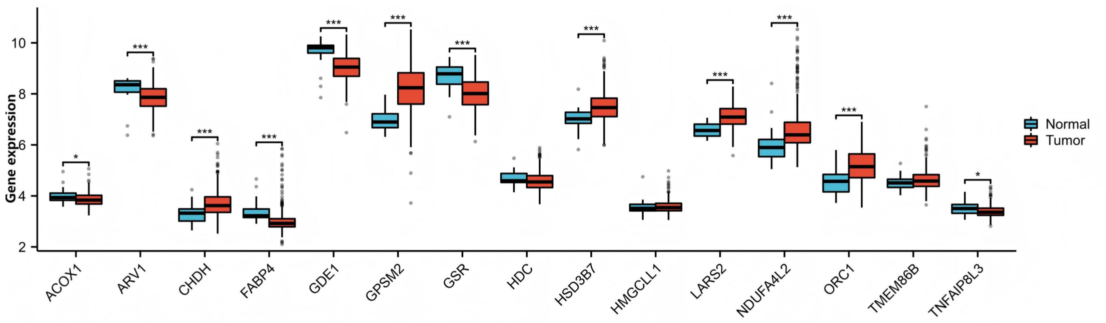

Figure S3. Gene Expression Levels of the 15 Prognosis-Related Genes in the GSE39582 Cohort. P-values are indicated as: ***P < 0.001, **P < 0.01, *P < 0.05

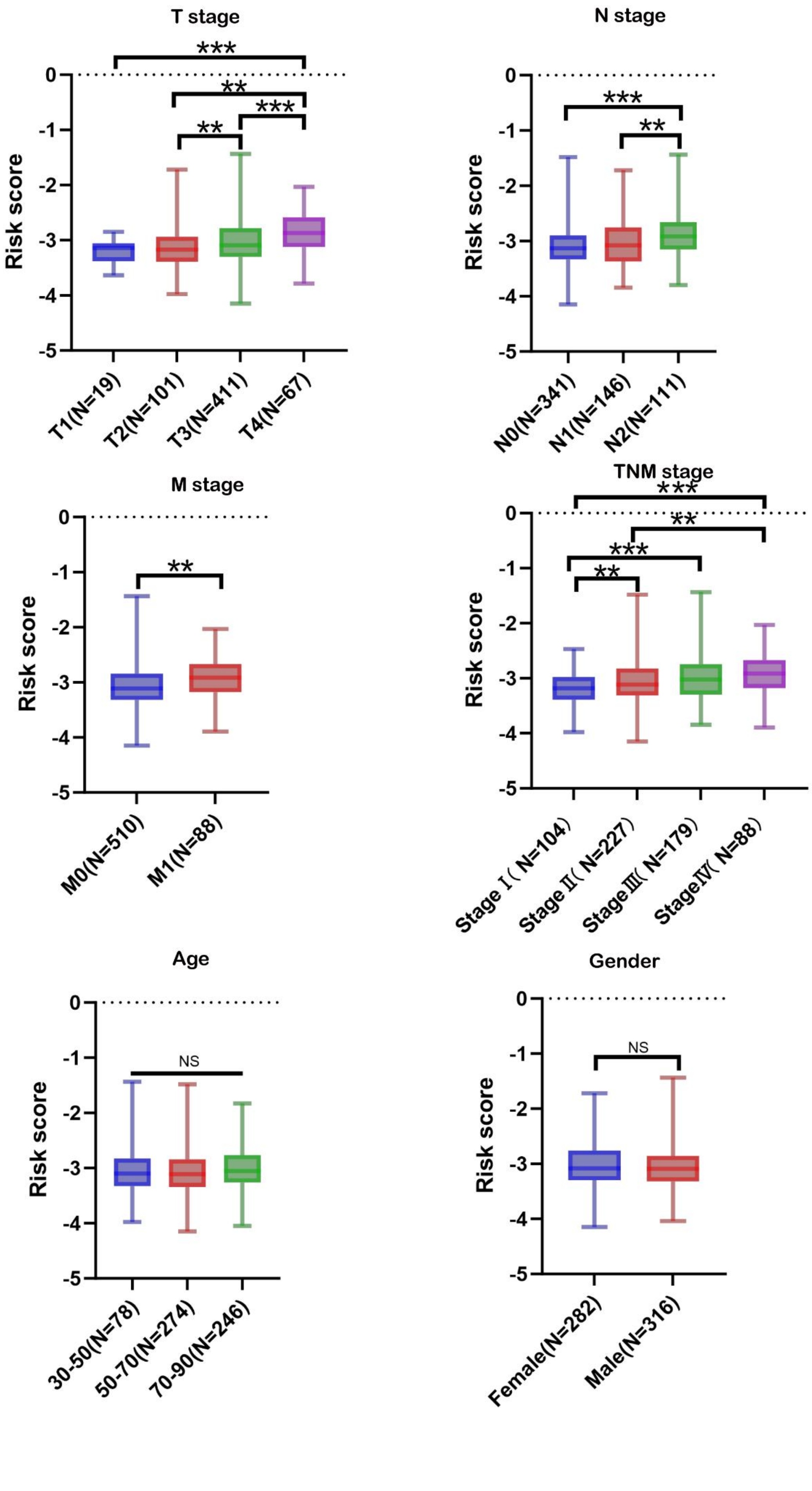
T stage
***
**
***
**
0
-1
-2
-3
-4
-5
Risk score
T1(N=19)
T2(N=101)
T3(N=411)
T4(N=67)
N stage
***
**
0
-1
-2
-3
-4
-5
Risk score
N0(N=341)
N1(N=146)
N2(N=111)
M stage
**
0
-1
-2
-3
-4
-5
Risk score
M0(N=510)
M1(N=88)
TNM stage
***
**
***
**
0
-1
-2
-3
-4
-5
Risk score
Stage Ⅰ(N=104)
Stage Ⅱ(N=227)
StageⅢ(N=179)
StageⅣ(N=88)
Age
NS
0
-1
-2
-3
-4
-5
Risk score
30-50(N=78)
50-70(N=274)
70-90(N=246)
Gender
NS
0
-1
-2
-3
-4
-5
Risk score
Female(N=282)
Male(N=316)

Figure S4. The Relationships between the Risk Score and Clinical Characteristics of COADREAD Patients. Age, Gender, T stage, N stage, M stage, Tumor stage. P-values are shown as: ns (not significant); *p < 0.05; **p < 0.01; ***p < 0.001.

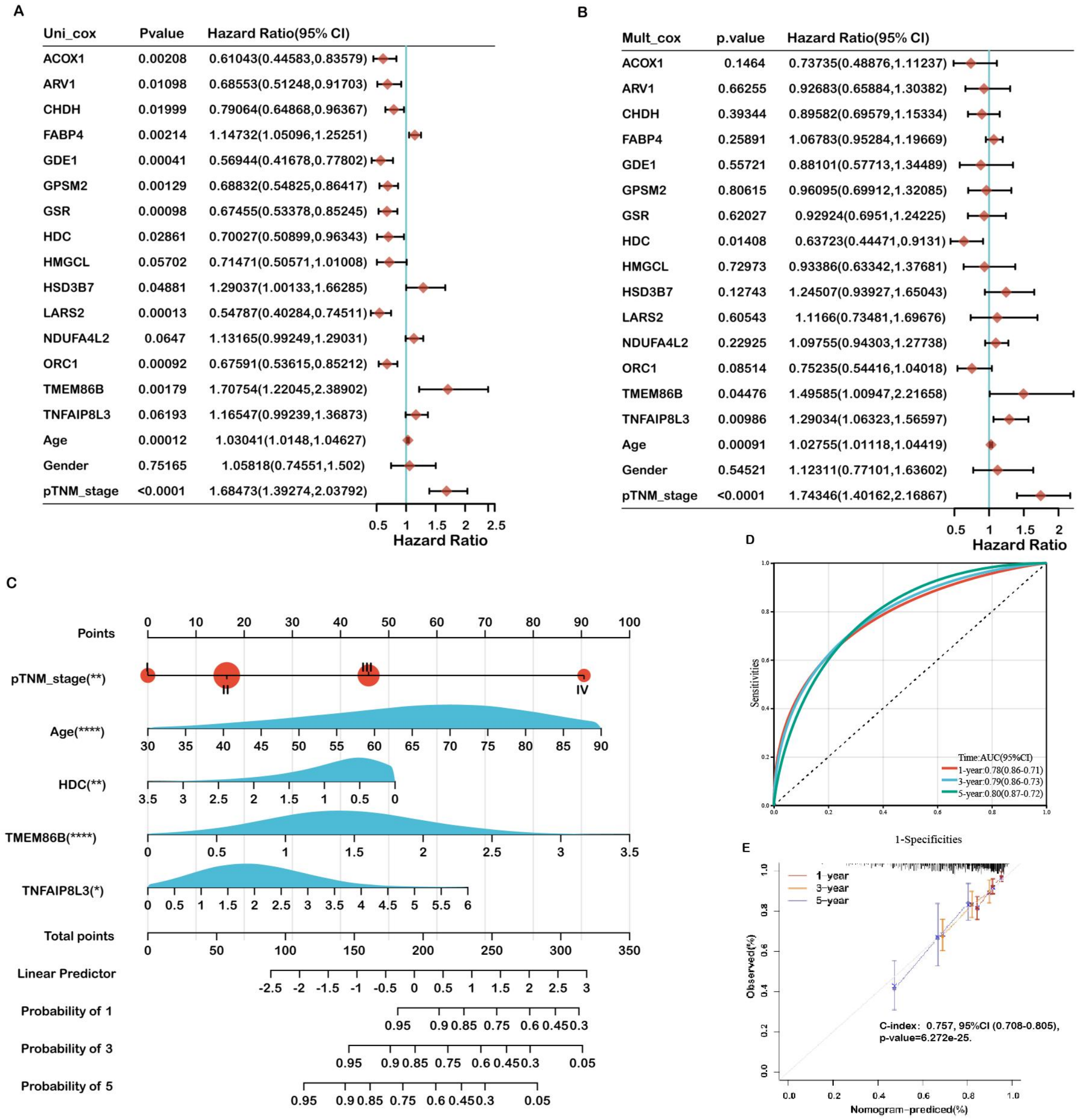

Figure S5. A Nomogram was Developed to Quantitatively Predict Patient Prognosis by Integrating Independent Prognostic Genes and Key Clinical Features

(A) Univariate Cox regression of prognosis-related genes associated with the prognosis of patients with COADREAD. (B) Multivariable Cox regression analysis revealing three independent prognostic biomarker genes associated with

the prognosis of patients with COADREAD. (C) A nomogram constructed based on three independent prognosis-related genes and related clinical characteristics. (D) Calibration charts predicting 1-, 3- and 5-year survival in TCGA dataset. The horizontal axis and vertical axis represent the predicted survival probability and the actual survival probability.

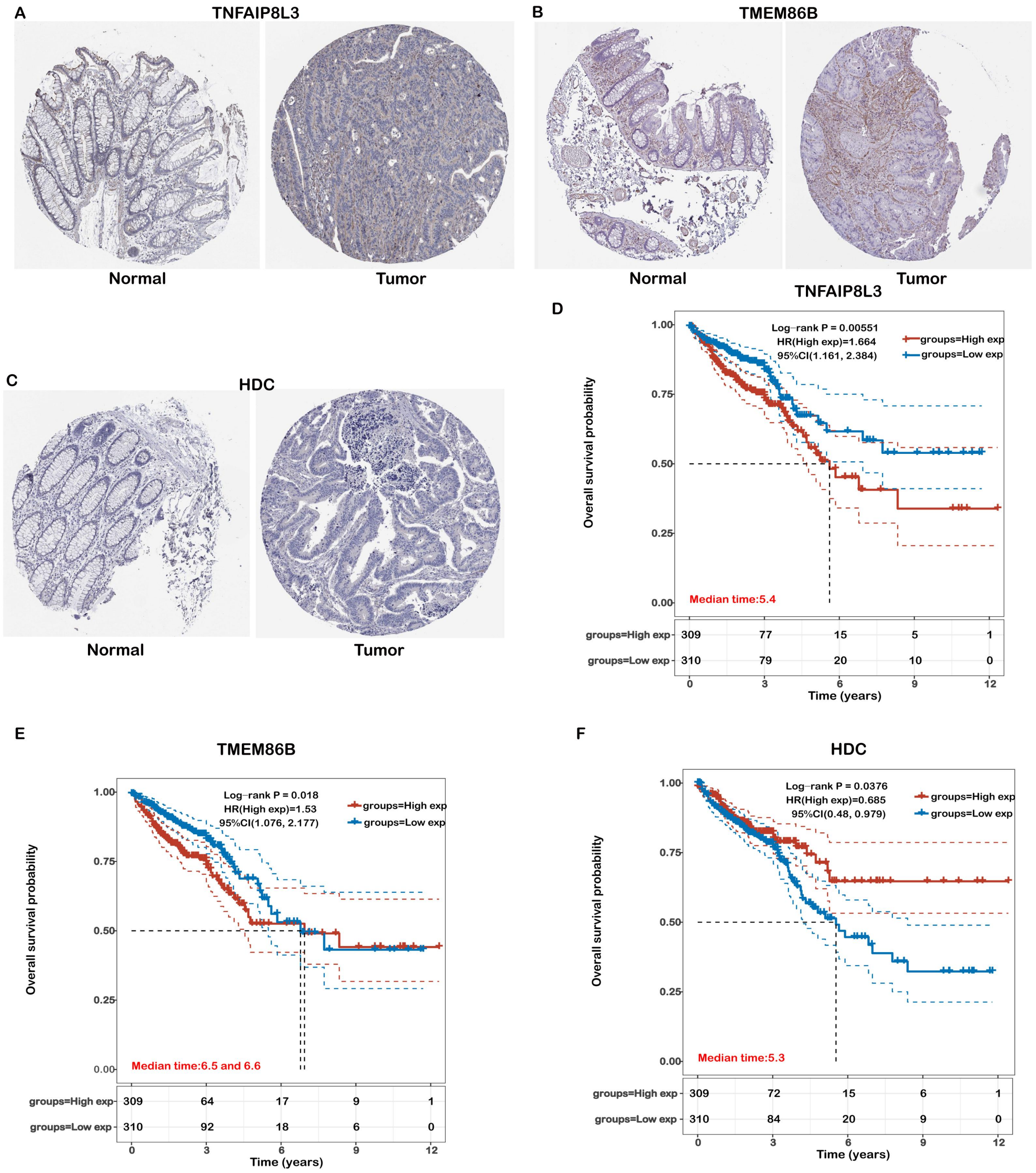

Figure S6. Protein Expressions of the Three Prognosis-Related Genes in CRC and Normal Tissues from the HPA Database and Kaplan–Meier Curves for OS

of Patients with High or Low Expression of Each Prognosis-Related Gene.(A) and (D) TNFAIP8L3, (B) and (E) TMEM86B, (C) and (F) HDC

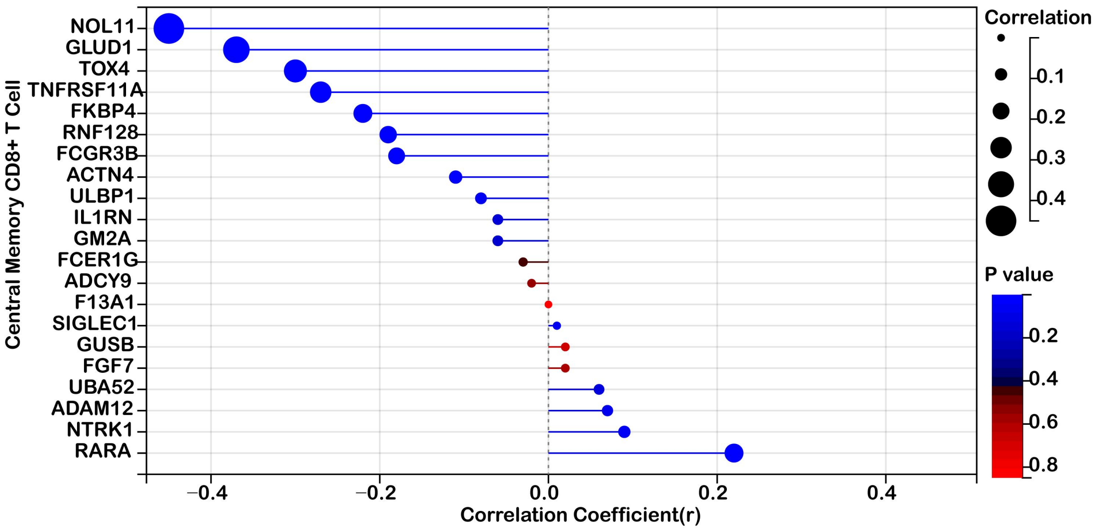

Figure S7. Analysis of the Correlation between the Risk Score and Central Memory CD8+ T Cell Signatures

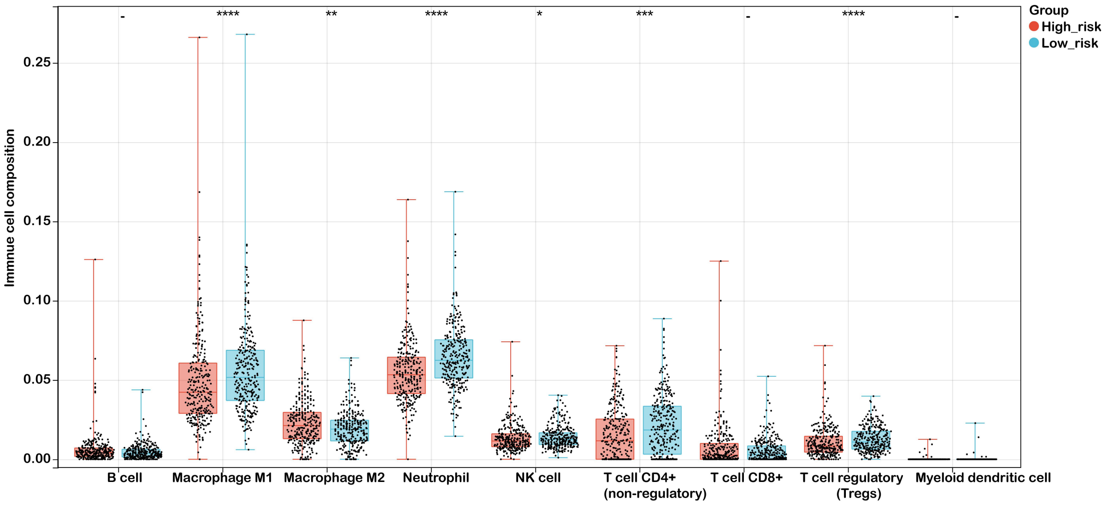

Figure S8. Immune QuanTIseq Analysis in Low-Risk and High-Risk Groups

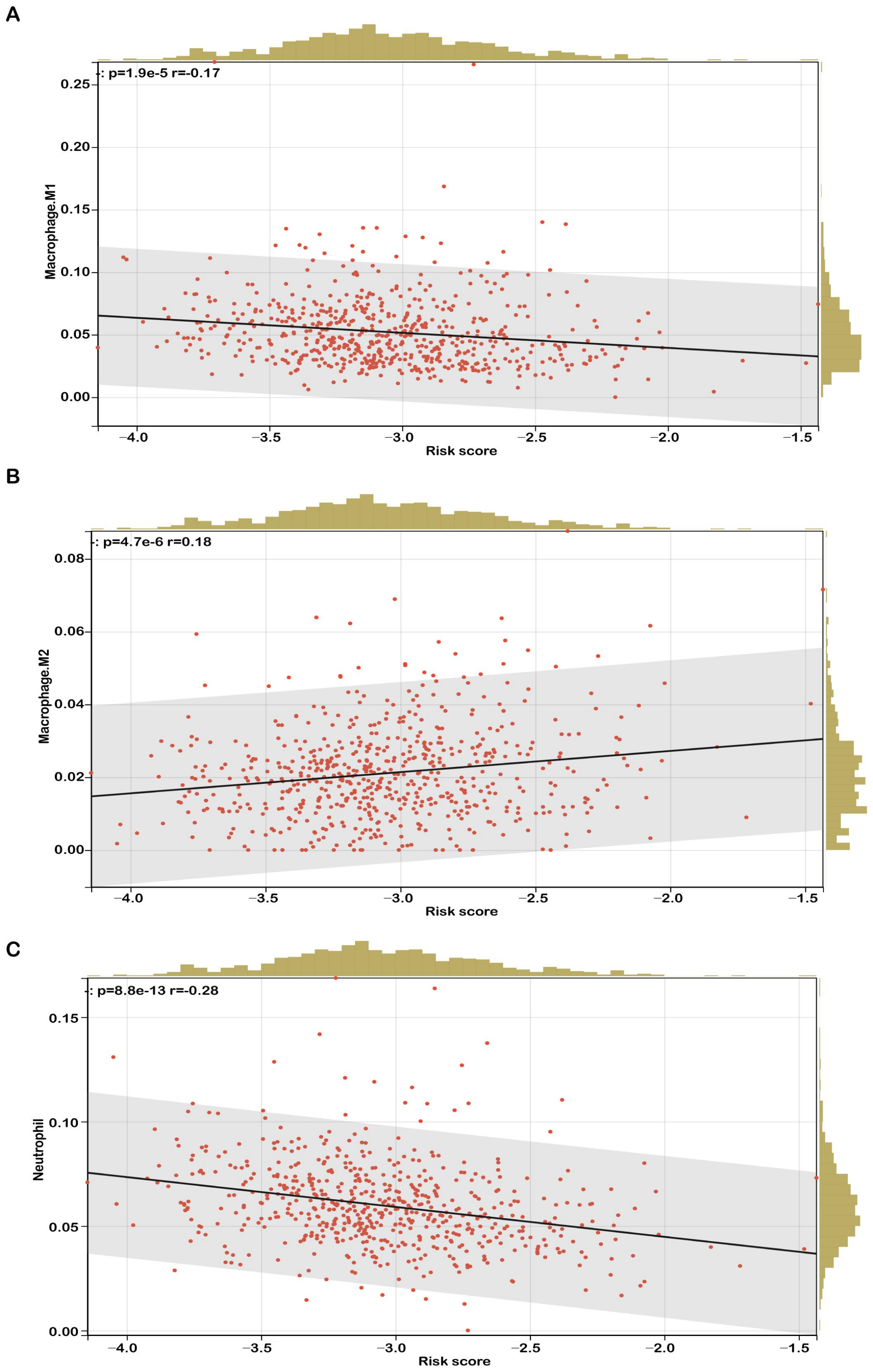

Figure S9. Correlation Analysis between the Risk Score and Immune Cells

(A) M1 macrophages (B) M2 macrophages (C) Neutrophils

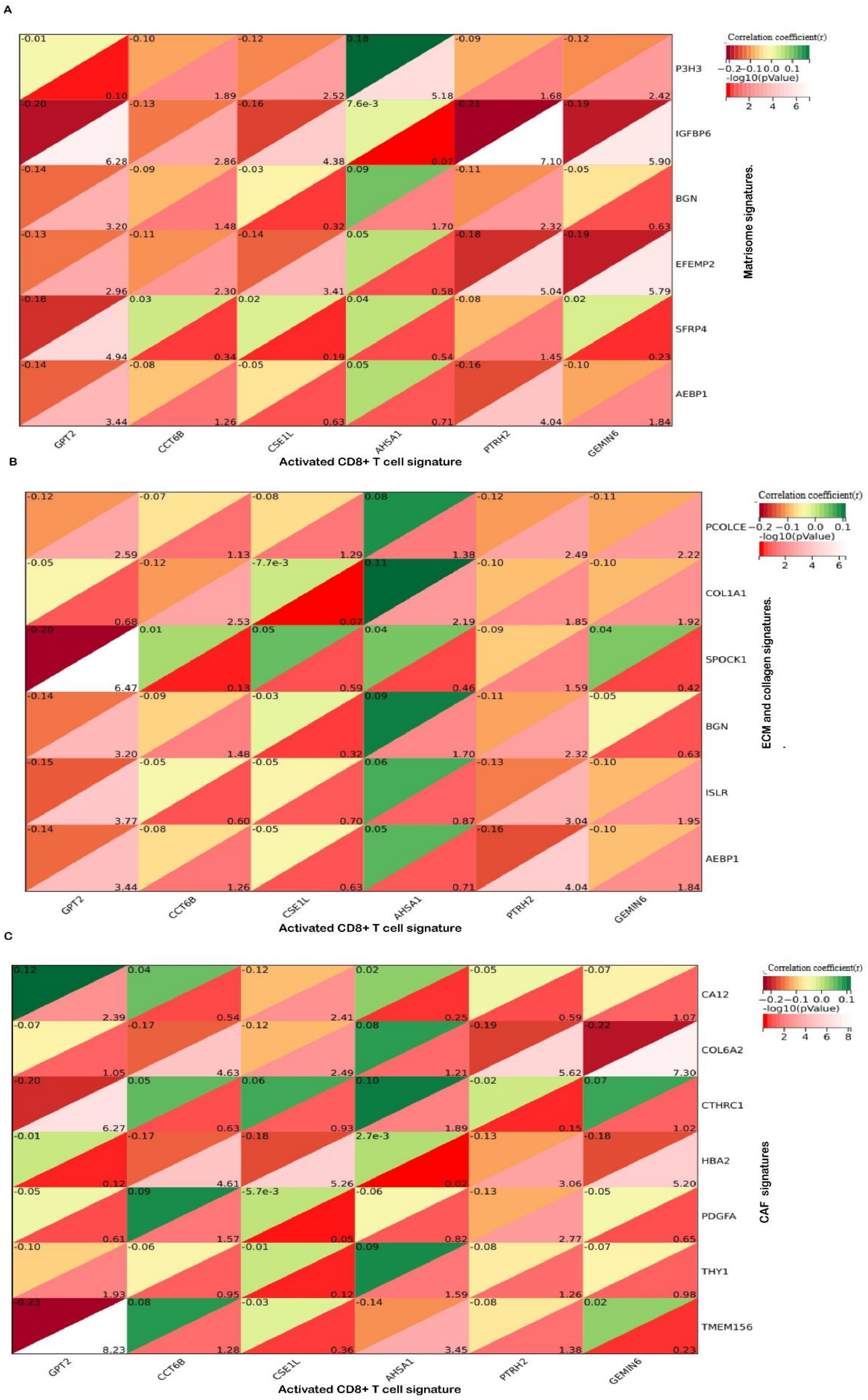
A
Correlation coefficient(r)
−0.2 −0.1 0.0 0.1
-log10(pValue)
2 4 6
-0.01 0.10 -0.10 1.89 -0.12 2.52 0.18 5.18 -0.09 1.68 -0.12 2.42
P3H3
-0.20 6.28 -0.13 2.86 -0.16 4.38 7.6e-3 0.07 -0.21 7.10 -0.19 5.90
IGFBP6
-0.14 3.20 -0.09 1.48 -0.03 0.32 0.09 1.70 -0.11 2.32 -0.05 0.63
BGN
-0.13 2.96 -0.11 2.30 -0.14 3.41 0.05 0.58 -0.18 5.04 -0.19 5.79
EFEMP2
-0.18 4.94 0.03 0.34 0.02 0.19 0.04 0.54 -0.08 1.45 0.02 0.23
SFRP4
-0.14 3.44 -0.08 1.26 -0.05 0.63 0.05 0.71 -0.16 4.04 -0.10 1.84
AEBP1
Matrisome signatures.
GPT2
CCT6B
CSE1L
AHSA1
PTRH2
GEMIN6
Activated CD8+ T cell signature
B
Correlation coefficient(r)
−0.2 −0.1 0.0 0.1
-log10(pValue)
2 4 6
-0.12 2.59 -0.07 1.13 -0.08 1.29 0.08 1.38 -0.12 2.49 -0.11 2.22
PCOLCE
-0.05 0.68 -0.12 2.53 -7.7e-3 0.07 0.11 2.19 -0.10 1.85 -0.10 1.92
COL1A1
-0.20 6.47 0.01 0.13 0.05 0.59 0.04 0.46 -0.09 1.59 0.04 0.42
SPOCK1
-0.14 3.20 -0.09 1.48 -0.03 0.32 0.09 1.70 -0.11 2.32 -0.05 0.63
BGN
-0.15 3.77 -0.05 0.60 -0.05 0.70 0.06 0.87 -0.13 3.04 -0.10 1.95
ISLR
-0.14 3.44 -0.08 1.26 -0.05 0.63 0.05 0.71 -0.16 4.04 -0.10 1.84
AEBP1
ECM and collagen signatures.
GPT2
CCT6B
CSE1L
AHSA1
PTRH2
GEMIN6
Activated CD8+ T cell signature
C
Correlation coefficient(r)
−0.2 −0.1 0.0 0.1
-log10(pValue)
2 4 6 8
0.12 2.39 0.04 0.54 -0.12 2.41 0.02 0.25 -0.05 0.59 -0.07 1.07
CA12
-0.07 1.05 -0.17 4.63 -0.12 2.49 0.08 1.21 -0.19 5.62 -0.22 7.30
COL6A2
-0.20 6.27 0.05 0.63 0.06 0.93 0.10 1.89 -0.02 0.15 0.07 1.02
CTHRC1
-0.01 0.12 -0.17 4.61 -0.18 5.26 2.7e-3 0.02 -0.13 3.06 -0.18 5.20
HBA2
-0.05 0.61 0.09 1.57 -5.7e-3 0.05 -0.06 0.82 -0.13 2.77 -0.05 0.65
PDGFA
-0.10 1.93 -0.06 0.95 -0.01 0.12 0.09 1.59 -0.08 1.26 -0.07 0.98
THY1
-0.23 8.23 0.08 1.28 -0.03 0.36 -0.14 3.45 -0.08 1.38 0.02 0.23
TMEM156
CAF signatures
GPT2
CCT6B
CSE1L
AHSA1
PTRH2
GEMIN6
Activated CD8+ T cell signature

Figure S10. Correlation Analysis between Activated CD8+ T Cell Signatures and TME Signatures. (A) Matrisome signatures (B) ECM and collagen signatures (C) CAF signatures.In the upper left triangle, red tones show positive correlations and yellow tones show negative ones. In the lower right triangle, lighter colors indicate smaller p-values and stronger statistical significance.

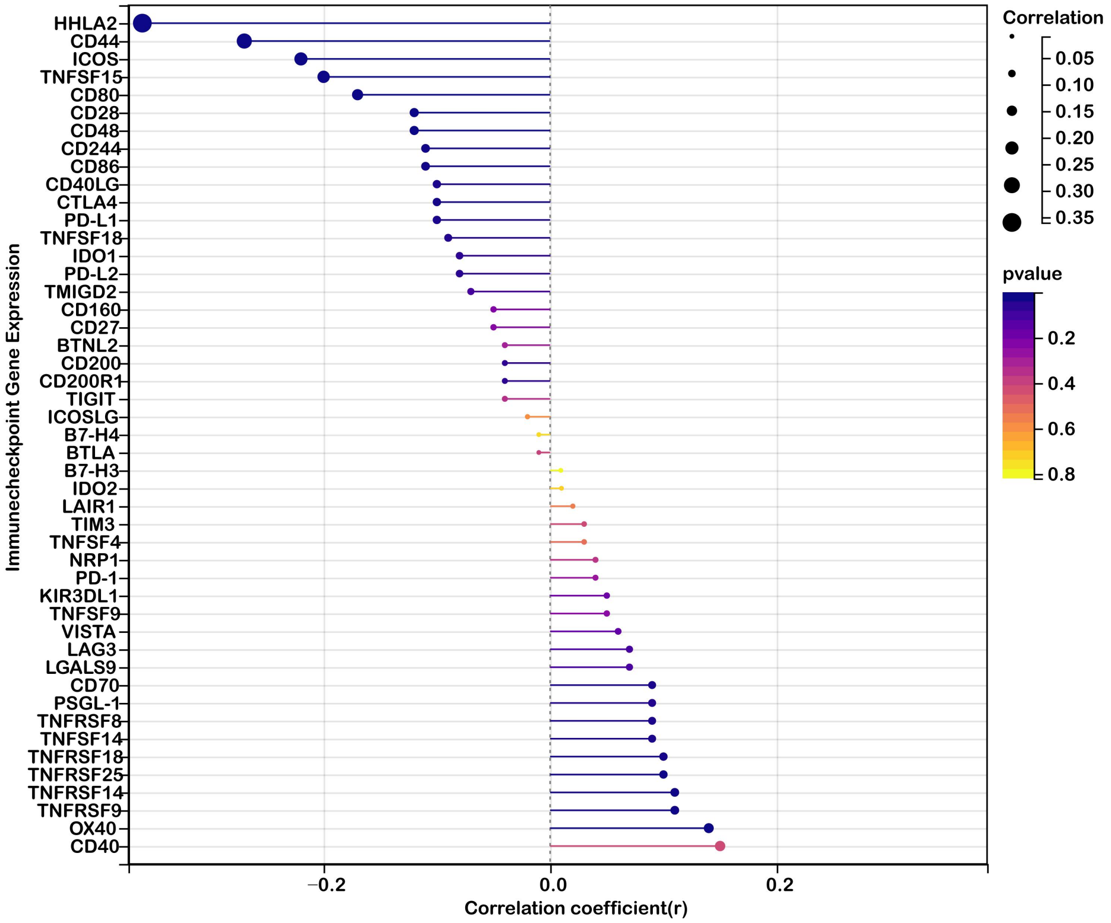

Figure S11. Examination of the Correlation between the Risk Score and Immune Checkpoint Expression

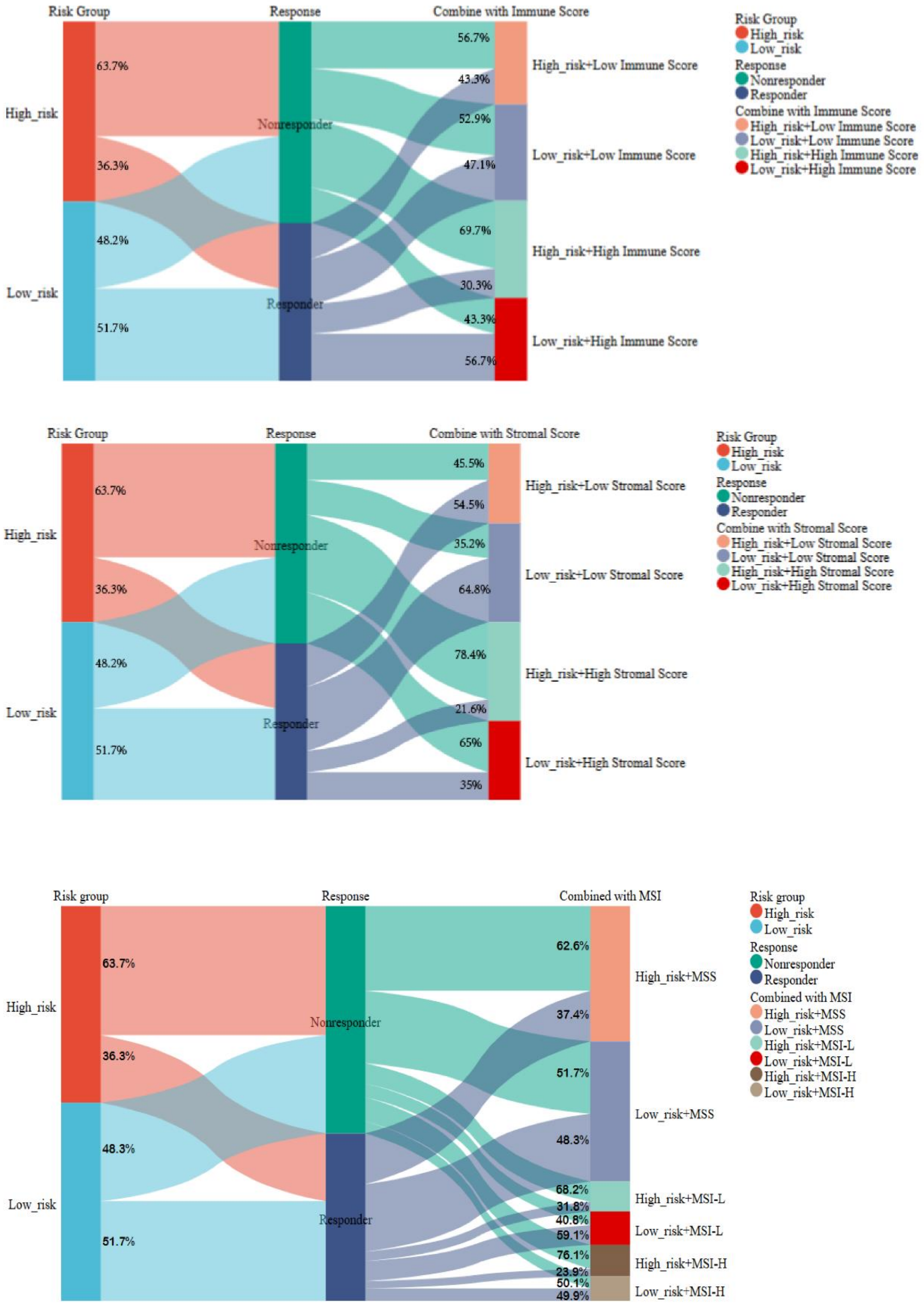

Figure S12. A Graphical Summary Illustrates that the Risk Score is a Potential Biomarker for Predicting the Benefits of Immune Therapies in COADREAD

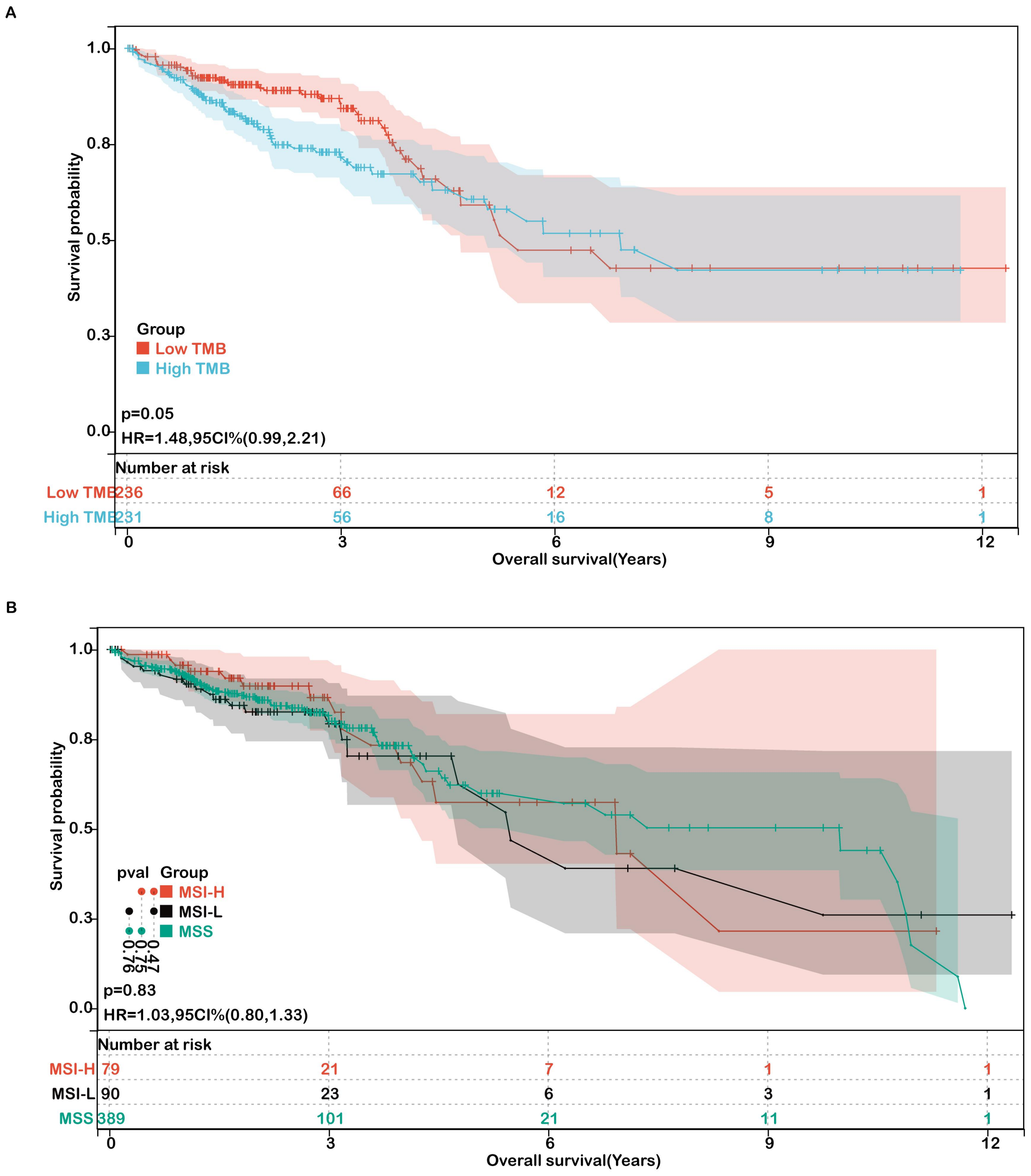

Figure S13.Mutation Status in High- and Low-Risk Groups in COADREAD (A) Kaplan – Meier curves of overall survival (OS) of patients in high- and low-TMB groups in the TCGA-COADREAD cohort. (B) Kaplan – Meier curves of OS of patients in MSS and MSI-H groups in the TCGA-COADREAD cohort.

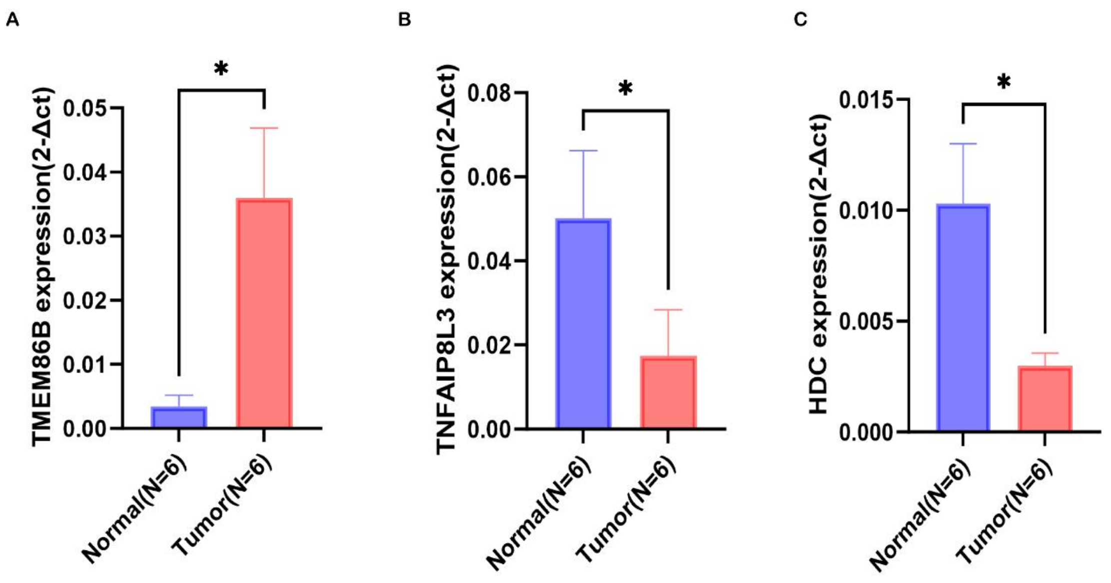

Figure. S14 Experimental verification of 3 genes expression in CRC. Expression of 3 genes in 6 paired CRC tissues and normal tissues was evaluated by qRT-PCR. (A) TMEM86B(B) TNFAIP8L3(C) HDC

Gene symbol	Gene ID	Full name	Location	Function of the encoded protein
HSD3B7	80270	Hydroxy-Delta-5-Steroid Dehydrogenase	Endoplasmic reticulum membrane	HSD3B7 encodes an enzyme which is involved in the initial stages of the synthesis of bile acids from cholesterol and a member of the short-chain dehydrogenase/reductase superfamily. The encoded protein is a membrane-associated endoplasmic reticulum protein which is active against 7-alpha hydrosylated sterol substrates. Mutations in this gene are associated with a congenital bile acid synthesis defect which leads to neonatal cholestasis, a form of progressive liver disease.
ORC1	4998	Origin Recognition Complex Subunit 1	Nucleus	Component of the origin recognition complex (ORC) that binds origins of replication. DNA-binding is ATP-dependent. The DNA sequences that define origins of replication have not been identified yet. ORC is required to assemble the pre-replication complex necessary to initiate DNA replication.
GPSM2	29899	G Protein Signaling Modulator 2	Cytosol	GPSM2 belongs to a family of proteins that modulate activation of G proteins, which transduce extracellular signals received by cell surface receptors into integrated cellular responses. The N-terminal half of this protein contains 10 copies of leu-gly-asn (LGN) repeat, and the C-terminal half contains 4 GoLoco motifs, which are involved in guanine nucleotide exchange. This protein may play a role in neuroblast division and in the development of normal hearing.
NDUFA4L2	56901	NADH dehydrogenase [ubiquinone] 1 alpha subcomplex subunit 4-like 2	Mitochondrion	Predicted to be integral component of membrane. Predicted to be part of mitochondrial respiratory chain complex IV
CHDH	55349	Choline Dehydrogenase,Mitochondrial	Mitochondrion	The protein encoded by this gene is a choline dehydrogenase that localizes to the mitochondrion. Variations in this gene can affect susceptibility to choline deficiency.
LARS2	23395	Leucyl-TRNA Synthetase 2	Mitochondrion	LARS2 catalyzes the attachment of leucine to its cognate tRNA
TMEM86B	255043	Lysoplasmalogenase;	Endoplasmic reticulum	TMEM86B catalyzes the hydrolysis of the vinyl ether bond of choline or ethanolamine lysoplasmalogens, forming fatty aldehyde and glycerophosphocholine or glycerophosphoethanolamine, respectively and is specific for the sn-2-deacylated (lyso) form of plasmalogen.
FABP4	2167	Fatty Acid Binding Protein 4	Cytosol	FABP4 encodes the fatty acid binding protein found in adipocytes. Fatty acid binding proteins are a family of small, highly conserved, cytoplasmic proteins that bind long-chain fatty acids and other hydrophobic ligands.
TNFAIP8L3	388121	3-Hydroxy-3-Methylglutaryl-CoA Lyase	Nucleus	TNFAIP8L3 predicted to enable phosphatidylinositol binding activity and phosphatidylinositol transfer activity. Predicted to be involved in several processes, including inositol lipid-mediated signaling; positive regulation of intracellular signal transduction; and positive regulation of phosphatidylinositol 3-kinase activity.
HMGCL	3155	Hydroxy-3-Methylglutaryl-CoA Lyase	Mitochondrion	The protein encoded by this gene belongs to the HMG-CoA lyase family. It is a mitochondrial enzyme that catalyzes the final step of leucine degradation and plays a key role in ketone body formation. Mutations in this gene are associated with HMG-CoA lyase deficiency
GDE1	51573	Glycerophosphodiester Phosphodiesterase 1	Plasma membrane	Predicted to enable glycerophosphodiester phosphodiesterase activity; glycerophosphoinositol glycerophosphodiesterase activity; and lysophospholipase activity. Predicted to be involved in N-acylethanolamine metabolic process; ethanolamine metabolic process; and phospholipid metabolic process.
ACOX1	51	Acyl-Coenzyme A Oxidase 1,Palmitoyl	Cytosol	The protein encoded by this gene is the first enzyme of the fatty acid beta-oxidation pathway, which catalyzes the desaturation of acyl-CoAs to 2-trans-enoyl-CoAs. It donates electrons directly to molecular oxygen, thereby producing hydrogen peroxide.
ARV1	64801	ARV1 Homolog,Fatty Acid Homeostasis Modulator	Endoplasmic reticulum	ARV1 plays a role as a mediator in the endoplasmic reticulum (ER) cholesterol and bile acid homeostasis and participates in sterol transport out of the ER and distribution into plasma membranes
HDC	3067	Histidine decarboxylase	Cytosol	HDC catalyzes the biosynthesis of histamine from histidine. Belongs to the group II decarboxylase family.
GSR	2936	Glutathione Reductase	Mitochondrion	GSR maintains high levels of reduced glutathione in the cytosol.

Table 1 The information of 15 prognosis-related genes

Characteristics	High_risk(N=299)	Low_risk(N=299)	Total(N=598)	Pvalue
Gender				0.06
Female	129(21.57%)	153(25.59%)	282(47.16%)	
Male	170(28.43%)	146(24.41%)	316(52.84%)	
Pathologic_stage				0.64
I	47(7.86%)	57(9.53%)	104(17.39%)	
II	114(19.06%)	113(18.90%)	227(37.96%)	
III	90(15.05%)	89(14.88%)	179(29.93%)	
IV	48(8.03%)	40(6.69%)	88(14.72%)	
Pathologic_T				0.36
T1	7(1.17%)	12(2.01%)	19(3.18%)	
T2	45(7.53%)	56(9.36%)	101(16.89%)	
T3	211(35.28%)	200(33.44%)	411(68.73%)	
T4	36(6.02%)	31(5.18%)	67(11.20%)	
Pathologic_N				0.51
N0	164(27.42%)	177(29.60%)	341(57.02%)	
N1	75(12.54%)	71(11.87%)	146(24.41%)	
N2	60(10.03%)	51(8.53%)	111(18.56%)	
Pathologic_M				0.42
M0	251(41.97%)	259(43.31%)	510(85.28%)	
M1	48(8.03%)	40(6.69%)	88(14.72%)	
Age				0.62
30-50	43(7.19%)	35(5.85%)	78(13.04%)	
50-70	135(22.58%)	139(23.24%)	274(45.82%)	
70-90	121(20.23%)	125(20.90%)	246(41.14%)	

Table 2 Clinical characteristics between low- and high-risk groups

Gene	Coefficient	Hazard Ratio (HR)	95% Confidence Interval	p-value
HSD3B7	0.1147	1.473	1.034 - 2.097	0.032
ORC1	-0.1300	0.546	0.380 - 0.785	0.001
GPSM2	-0.0259	0.677	0.476 - 0.961	0.029
NDUFA4L2	0.0120	1.649	1.158 - 2.348	0.006
CHDH	-0.0317	0.598	0.419 - 0.854	0.005
LARS2	-0.1246	0.587	0.410 - 0.841	0.004
TMEM86B	0.2367	1.530	1.076 - 2.177	0.018
FABP4	0.0779	1.720	1.202 - 2.459	0.003
TNFAIP8L3	0.0759	1.664	1.161 - 2.384	0.006
HMGCL	-0.0059	0.690	0.485 - 0.982	0.039
GDE1	-0.1341	0.650	0.453 - 0.932	0.019
ACOX1	-0.1125	0.661	0.464 - 0.943	0.022
ARV1	-0.0459	0.505	0.353 - 0.723	<0.001
HDC	-0.2789	0.685	0.480 - 0.979	0.038
GSR	-0.1676	0.509	0.353 - 0.734	<0.001

Table 3: Coefficients and Hazard Ratios of the 15-Gene Mitochondrial Metabolic Signature

Gene	Forward Primer (5′→3′)	Reverse Primer (5′→3′)
18S rRNA	ACCGCAGCTAGGAATAATGGA	CAAATGCTTTCGCTCTGGTC
TMEM86B	CACCTCCTCTACGTCTGGG	GCAGGACCATATCCGGCTC
HDC	ATGCACGCCTACTACCCAG	CAGTCCATGACGTTCATCTCC
TNFAIP8L3	AAGCACACTGGTTTCCACACT	TGGGTCCCTGCATATCCGTT

Table 4. Primer sequences used for qRT-PCR
